# Polymorphisms of *ESR1, UGT1A1, HCN1, MAP3K1* and *CYP2B6* are associated with the prognosis of hormone receptor-positive early breast cancer

**DOI:** 10.18632/oncotarget.14995

**Published:** 2017-02-02

**Authors:** Sung-Hsin Kuo, Shi-Yi Yang, San-Lin You, Huang-Chun Lien, Ching-Hung Lin, Po-Han Lin, Chiun-Sheng Huang

**Affiliations:** ^1^ Department of Oncology, National Taiwan University Hospital and National Taiwan University College of Medicine, Taipei, Taiwan; ^2^ Department of Pathology, National Taiwan University Hospital and National Taiwan University College of Medicine, Taipei, Taiwan; ^3^ Department of Medical Genetics, National Taiwan University Hospital and National Taiwan University College of Medicine, Taipei, Taiwan; ^4^ Department of Surgery, National Taiwan University Hospital and National Taiwan University College of Medicine, Taipei, Taiwan; ^5^ Graduate Institute of Oncology, National Taiwan University College of Medicine, Taipei, Taiwan; ^6^ Cancer Research Center, National Taiwan University College of Medicine, Taipei, Taiwan; ^7^ National Taiwan University Cancer Center, National Taiwan University College of Medicine, Taipei, Taiwan; ^8^ Graduate Institute of Epidemiology, College of Public Health, National Taiwan University, Taipei, Taiwan; ^9^ School of Medicine, College of Medicine, Fu-Jen Catholic University, New Taipei, Taiwan; ^10^ Big Data Research Center, Fu-Jen Catholic University, New Taipei, Taiwan

**Keywords:** genetic polymorphism, GWAS, breast cancer, prognostic factor, survival

## Abstract

In this study, we investigated whether single nucleotide polymorphisms (SNPs) identified by genome-wide association study (GWAS) (*MAP3K1*, *FGFR2*, *TNRC9*, *HCN1*, and *5p12*), and SNPs involved in the metabolism of estrogen (*CYP19, COMT*, *ESR1*, and *UGT1A1*), tamoxifen (*CYP2C9*, *CYP2C19, CYP3A5*, and *CYP2D6*), and chemotherapeutic agents (*ABCB1, ALDH3A1*, and *CYP2B6*) are associated with the prognoses of 414 hormone receptor (HR)-positive early breast cancers with negative or 1 to 3 nodal metastases. At a median follow-up period of 10.6 years, 363 patients were alive, and 51 (12.3%) had died. Multiple-adjusted hazard ratios (aHRs) and the corresponding 95% confidence intervals for distant disease-free survival (DDFS), disease-free survival (DFS), and overall survival (OS) in association with the genotypes of 34 SNPs from the above-mentioned 16 genes were evaluated, using the stepwise selection Cox model. We found that the SNP, *ESR1-*codon325 rs1801132 (G/G+G/C), was associated with a longer DDFS, whereas *UGT1A1* rs4148323 (A/A+A/G), and *HCN1* rs981782 (A/A+A/C) were significantly associated with poorer DDFS. *MAP3K1* rs889312 (C/C) and *CYP2B6* rs3211371 (T/C) were significantly associated with poor DFS, DDFS and OS. Among premenopausal women, *MAP3K1* rs889312 (C/C), *CYP2B6* rs3211371 (T/C), *CYP2B6* rs4802101 (T/T), *ABCB1* rs2032582 (C/C), and *ALDH3A1* rs2231142 (G/G) were significantly associated with poor DDFS, DFS, or OS. Our results provide additional evidence that genetic polymorphisms observed in SNPs are associated with the prognoses of patients with HR-positive breast cancers; this may indicate different treatment strategies for these patients.

## INTRODUCTION

Postoperative adjuvant systemic therapy is considered to be an integral component of the management of primary breast cancer [1, 2]. The decision to give adjuvant chemotherapy is based on prognostic and predictive factors, such as age, axillary lymph node status, histologic grade, tumor size, and hormone receptor (HR) status [1, 2]. Several multiple gene assays have been demonstrated to predict the survival of HR-positive patients, and help physicians and patients to decide whether to administer adjuvant chemotherapy [3–6]. However, these assays only test the alteration of gene expression from tumor tissues but do not test the underlying genetic variations of the patient [3–6].

**Figure 1:**
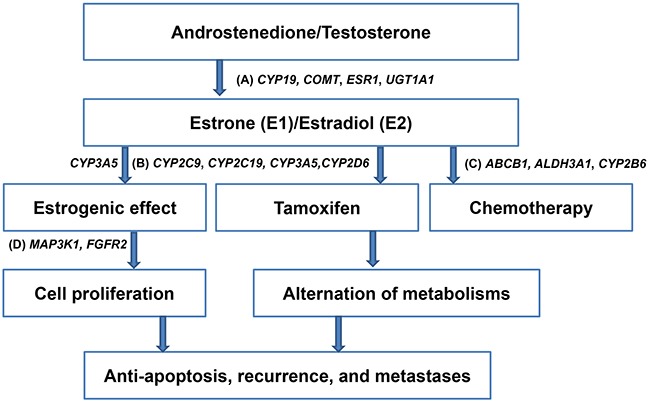
Schema illustrating single nucleotide polymorphisms that involved in the metabolism of estrogen, tamoxifen, and chemotherapeutic agents, and cell proliferation of hormone receptor-positive breast cancer **A**. Candidate genes involved in the metabolism of estrogen, such as *CYP19*, *COMT*, *ESR1*, *UGT1A1*, and *CYP3A5* **B**. Candidate genes involved in the metabolism of tamoxifen, such as *CYP2C9*, *CYP2C19, CYP3A5*, and *CYP2D6* **C**. Candidate genes involved in the metabolism of chemotherapeutic agents, such as *ABCB1, ALDH3A1*, and *CYP2B6* **D**. Genome-wide association studies-derived genes involved in the cell proliferation of breast cancer cells, such as *MAP3K1* and *FGFR2*.

Our and others’ studies have previously demonstrated that patients with different genotypes of single nucleotide polymorphisms (SNPs) of estrogen and tamoxifen metabolizing genes, such as *CYP19*, *COMT, CPY2D6*, and *SULT1A1*, may carry different responses to anti-estrogen treatment and hence have different outcomes [7–11]. In addition to these candidate genes, SNPs identified from genome-wide association studies (GWAS) have been found to be associated with breast cancer risk [12–16] and survival [17, 18]. Taken together, we hypothesized that host factors, as shown by SNPs identified from GWAS and SNPs of genes involved in the metabolism of estrogen, tamoxifen, and chemotherapeutic agents (Figure 1), may influence the effect of adjuvant treatment, and thus the survival of breast cancer patients.

In the present study, we aimed to investigate whether these 34 SNPs, which included GWAS-identified genes, such as, *MAP3K1* rs889312, *FGFR2* rs2981582, *TNRC9* (or *TOX3*) rs3803662, *HCN1* rs981782, and *5p12* rs10941679 and rs4415084; candidate genes involved in the metabolism of estrogen, such as, *CYP19* (TTTA)n, rs4646, rs1065779, rs1870050, and rs700519, *COMT* rs4680, *ESR1* rs3020314, rs3020396, rs2982684, rs1801132, rs2234693, and rs2046210, and *UGT1A1* rs4148323; the metabolism of tamoxifen, such as *CYP2C9* rs1057910, *CYP2C19* rs4244285 and rs4986893*, CYP3A5* rs776746, and *CYP2D6* rs16947, rs1065852, rs28371725 and rs3892097; and the metabolism of chemotherapeutic agents, such as *ABCB1* rs1128503, rs2032582, and rs1045642*, ALDH3A1* rs2231142 and rs2228100, and *CYP2B6* rs4802101 and rs3211371 are associated with the prognoses, including the distant disease-free survival (DDFS), disease-free survival (DFS) and overall survival (OS), of early-stage HR-positive breast cancers with negative or 1 to 3 nodal metastases.

## RESULTS

### Clinicopathologic features of hormone receptor-positive patients

Four hundred and fourteen patients were included in the study. As shown in Table 1, the median age was 48 years (range 23-81 years) and 251 subjects were premenopausal and 163 were postmenopausal. The clinicopathologic characteristics and treatments are also listed in Table 1. As shown in Table 1, histologic subtypes of our breast cancer included infiltrating ductal carcinoma (IDC) (n=349, 84.3%), infiltrating lobular carcinoma (ILC) (n=16, 3.9%), medullary carcinoma (n=3, 0.7%), ductal carcinoma *in situ* (DCIS) with microinvsion (n=14, 3.4%), tubular carcinoma (n=4, 0.9%), mucinous carcinoma (n=23, 5.6%), and papillary carcinioma (n=5, 1.2%). Briefly, 384 (92.8%) of 414 patients received adjuvant hormonal therapy with tamoxifen, and 30 (7.2%) patients received ovarian ablation or a luteinizing hormone-releasing hormone agonist with or without tamoxifen. Because aromatase inhibitor was not reimbursed by the National Health Insurance for our patients treated with multimodality treatment between January 1, 1994 and June 30, 2006, none of them received aromatase inhibitor. Two hundred and ninety-six patients (71.5%) were LN-negative, whereas 118 patients (28.5%) had 1 to 3 LN metastases. One hundred and sixty-three (39.4%) did not receive chemotherapy, and 251 (60.6%) received standard adjuvant chemotherapy (Table 1). Furthermore, 308 (74.4%) of 414 patients were positive for both ER and PR.

**Table 1:** Demographics and clinical characteristics of 414 hormone receptor (HR)-positive breast cancer patients

Characteristic	HR(+) (N=414)
Age (years)	
Median (range)	48 (23-81)
LN	
0	296 (71.5)
1-3	118 (28.5)
Menopausal status	
Premenopausal	251 (60.6)
Postmenopausal	163 (39.4)
Pathology	
Infiltrating ductal carcinoma	349 (84.3)
Infiltrating lobular carcinoma	16 (3.9)
Medullary carcinoma	3 (0.7)
DCIS+Microinvasion	14(3.4)
Tubular carcinoma	4 (0.9)
Mucinous carcinoma	23 (5.6)
Papillary carcinoma	5 (1.2)
Grade	
I	171 (41.3)
II	187 (45.2)
III	56 (13.5)
Tumor size (cm)	
<=2	219 (53.2)
>2-5	193 (46.8)
Missing	2
Hormone receptor status	
ER (+) PR (+)	308 (74.4)
ER (+) PR (-)	64 (15.5)
ER (-) PR (+)	42 (10.1)
Adjuvant hormone therapy	
Tamoxifen	384 (92.8)
Others*	30 (7.2)
Adjuvant chemotherapy	
CT**	251 (60.6)
No CT	163 (39.4)

Abbreviations: LN, lymph node; DCIS, ductal carcinoma *in situ*; ER, estrogen receptor; PR, progesterone receptor; CT, chemotherapy

*: Ovarian ablation or luteinizing hormone-releasing hormone

** CT regimen: CEF (117 cases, 46.6%), CMF (97 cases, 38.6%), AC (26 cases, 10.4%), and AC+paclitaxel (11 cases, 4.4%)

C, cyclophosphamide; E, epirubicin; F., 5-FU; M, methotrexate; A, adriamycin.

The median follow-up period was 10.6 years (7.2% of patients were followed-up for more than 15 years, and 5.1% for less than 5 years); by the end of the follow-up period, 51 (12.3%) patients had died (43 [84.3%] due to breast cancer, and 8 [15.7%] due to causes not related to breast cancer), and 363 remained alive. Among the patients who had died due to causes that were not related to breast cancer, 1 experienced senility without the presence of psychosis; 1 had diabetes; 2 had malignancies other than breast cancers; 1 had lymphoma; 1 had a urinary tract infection that was accompanied by sepsis; 1 had a malignant neoplasm of the urethra; and 1 had coronary atherosclerosis. Due to the limited proportion of deaths that were not related to breast cancers, we believe that these were not confounding factors in our results.

### SNPs associated with good survival

Using the stepwise selection multiple Cox model analyses (adjusted multiple SNPs and clinicopathologic features), we revealed that *ESR1* codon325 rs1801132 (G/G/+G/C vs. C/C) was the only SNP significantly associated with good survival in all women (DDFS, *P* = 0.05) (Table 2).

**Table 2:** Multiple stepwise selection cox model of the predictors of survival in hormone receptor-positive early breast cancer patients

Total patients	DDFS		DFS		OS	
	**aHR (95%CI)**	***P***	**aHR (95%CI)**	***P***	**aHR (95%CI)**	**P**
ESR1 codon325 rs1801132 (G/G/+G/C vs. C/C)	0.6 (0.3-1.0)	0.05				
UGT1A1 rs4148323 (A/A+A/G vs. G/G)	1.9 (1.1-3.1)	0.02				
CYP2B6 rs3211371 (T/C vs. C/C)	322.2 (25.2-4113.7)	<0.0001	140.0(14.3-1375.2)	<0.0001	129.1(14.0-1190.1)	<0.0001
MAP3K1 rs889312 (C/C vs. C/A+A/A)	2.3 (1.4-3.8)	0.002	2.1 (1.3-3.4)	0.001	2.1 (1.1-3.8)	0.02
HCN1 rs981782 (A/A+A/C vs. C/C)	4.6 (1.1-19.1)	0.04				
ER (-) PR (+) vs. ER (+) PR (-)/ER (+) PR (+)			2.0 (1.1-3.8)	0.02	2.3 (1.1-5.0)	0.03
**Premenopausal patients**						
CYP2B6 rs4802101 (T/T vs. C/C+C/T)	3.3 (1.4-6.9)	0.004				
CYP2B6 rs3211371 (T/C vs. C/C)	18.0 (2.0-165.2)	0.01	118.0 (10.3-1349.6)	0.0001	70.5 (6.4-779.6)	0.0005
MAP3K1 rs889312 (C/C vs. C/A+A/A)	2.4 (1.3-4.4)	0.007	2.0 (1.1-3.4)	0.02		
Pathologic status of grade III vs. grade I+II					1.8 (1.1-3.0)	0.03
ABCB1 rs2032582 (C/C vs. C/T+T/T)					3.4 (1.0-11.3)	0.05
ALDH3A1 rs2231142 (G/G vs. G/T+T/T)			0.6 (0.3-1.0)	0.05		
ER (-) PR (+) vs. ER (+)PR (-)/ER (+) PR (+)			2.2 (1.0-4.5)	0.04		
**Postmenopausal patients**						
	without any significant markers				without any significant markers	
**Patients receiving adjuvant chemotherapy (total women)**						
MAP3K1 rs889312 (C/C vs. C/A+A/A)	2.0 (1.0-3.8)	0.04	2.3 (1.2-4.1)	0.008		
**Patients receiving** **adjuvant hormonal therapy alone (total women)**						
UGT1A1 rs4148323 (A/A+A/G vs. G/G)	2.9 (1.2-6.7)	0.01				
CYP2B6 rs3211371 (T/C vs. C/C)	68.6 (6.7-697.4)	0.0004	126.5 (7.9-2022.4)	0.0006	297.3 (16.3-5420.9)	0.0001
Pathologic status of grade III vs. grade I+II					2.2 (1.1-4.6)	0.03
ESR1_pvuII rs2234693 (C/C+C/T vs. T/T)					0.3 (0.1-0.8)	0.01
MAP3K1 rs889312 (C/C vs. C/A+A/A)					3.0 (1.2-7.8)	0.02

Abbreviation: DDFS, distant disease-free survival; DFS, disease-free survival; OS, overall survival; aHR, adjusted hazard ratios.

### SNPs associated with poor survival

In multiple stepwise selection Cox model analyses, SNPs including *UGT1A1* rs4148323*, CYP2B6* rs3211371*, MAP3K1* rs889312*, HCN1* rs981782*, CYP2B6* rs4802101 and *ABCB1* rs2032582 were associated with poor survival (Table 2).

**Figure 2:**
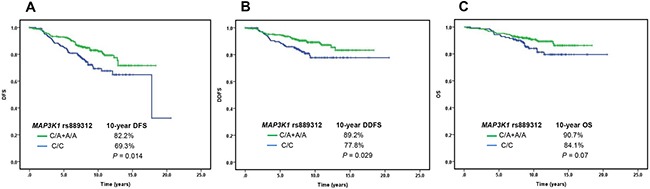
The association between single nucleotide polymorphisms of *MAP3K1* rs889312 and prognosis of hormone receptor-positive early-stage breast cancer **A**. Disease-free survival (DFS)) **B**. Distant disease-free survival (DDFS) **C**. overall survival (OS).

Among them, *CYP2B6* rs3211371 (*P* < 0.0001 for DDFS, DFS, and OS) and *MAP3K1* rs889312 (*P* = 0.002 for DDFS, *P* = 0.001 for DFS, and *P* = 0.02 for OS) were associated with poor survival for all women; and these two SNPs were predominantly associated with premenopausal women (*CYP2B6* rs3211371, *P* = 0.01 for DDFS, *P* = 0.0001 for DDFS, and *P* = 0.0005 for OS; *MAP3K1* rs889312, *P* = 0.007 for DDFS and *P* = 0.02 for DFS), but not associated with postmenopausal women. As shown in Table 3, patients with *CYP2B6* rs3211371 (T/C) had significantly poorer DDFS, DFS and OS than those with *CYP2B6* rs3211371 (C/C). Furthermore, patients with *MAP3K1* rs889312 (C/C) had significantly poorer DDFS and DFS, and a poorer OS than those with *MAP3K1* rs889312 (C/A+A/A) (Table 3 and Figure 2).

**Table 3:** Proportion of 5-year and 10-year survival according to SNPs of *CYP2B6* rs3211371 and *MAP3K1* rs889312

Survival rate	Genotype	DDFS		DFS		OS	
		**5-year (%)**	**10-year (%)**	**5-year (%)**	**10-year (%)**	**5-year (%)**	**10-year (%)**
***CYP2B6*** **rs3211371**	**T/C**	0	0	0	0	0	0
	**C/C**	91.91.	82.1	90.4	77.9	95.1	88.8
	***P*-value**	< 0.01		< 0.01		< 0.01	
***MAP3K1*** **rs889312**	**C/C**	88.9	77.8	85.3	69.3	94.3	84.1
	**C/A+A/A**	94.7	89.2	92.9	82.2	95.2	90.7
	***P*-value**	0.029		0.014		0.07	

Abbreviation: SNP, single nucleotide polymorphisms; DDFS, distant disease-free survival; DFS, disease-free survival; OS, overall survival.

Three other SNPs (*CYP2B6* rs4802101, *P* = 0.004 for DDFS; *ABCB1* rs2032582, *P* = 0.05 for OS; *ALDH3A1* rs2231142, *P* = 0.05 for DFS) were only associated with the survival of premenopausal women, whereas another 2 SNPs (*UGT1A1* rs4148323, *P* = 0.02 for DDFS; *HCN1* rs981782, *P* = 0.04 for DDFS) were associated with all women but not associated with premenopausal women or postmenopausal women.

In addition to the aforementioned SNPs, multiple Cox model analyses of the associations of prognosis with individual genotypes, adjusted by the clinicopathologic characteristics listed in Table 1 but not by other SNPs (Supplementary Table 1) showed that 7 other SNPs (*CYP19* rs4646*, CYP19* rs1870050, *CYP19* rs700519, *COMT* rs4680, CYP2D6*10, *FGFR2* rs2981582, and *ABCB1* rs1128503) and one *CYP19* (TTTA)n were associated with poor survival. Among them, *CYP19* (TTTA)n, *CYP19* rs1870050, *CYP19* rs700519, and *FGFR2* rs2981582) were associated with all women (Supplementary Table 1). Among these, 4 SNPs (*CYP19* rs4646, *CYP19* rs1870050, *COMT* rs4680, and *ABCB1* rs1128503) were predominantly associated with premenopausal women. The CYP2D6*10 (intermediate metabolizer phenotype) was the only SNP associated with the survival of postmenopausal women, but the significance of the CYP2D6*10 (intermediate metabolizer phenotype) was lost when all other SNPs were analyzed in the multiple stepwise selection Cox model (Table 2).

### SNPs associated with poor or good survival in patients with adjuvant hormonal therapy alone

Among women with adjuvant hormonal therapy alone (without adjuvant chemotherapy), indicating their good prognosis, one SNP (*ESR1*_pvuII rs2234693, *P* = 0.01 for OS) was associated with good survival by multiple stepwise selection Cox model. Furthermore, three SNPs (*UGT1A1* rs4148323, *P* = 0.01 for DDFS; *CYP2B6* rs3211371, *P* = 0.0004 for DDFS, *P* = 0.0006 for DFS, and *P* = 0.0001 for OS; *MAP3K1* rs889312, *P* = 0.02 for OS) were associated with poor prognosis for women without adjuvant chemotherapy (Table 2).

Also shown in Supplementary Table 1 (the associations of prognosis with individual genotypes, adjusted by conventional prognostic factors but not by other SNPs, using the multiple Cox model), *ESR1* codon325 rs1801132 were significantly associated with a better DDFS (*P* = 0.03) for patients not receiving adjuvant chemotherapy. Four SNPs (CYP19_(TTTA)n, *COMT* rs4680, *ABCB1* rs1128503 and *FGFR2* rs2981582) were significantly associated with poor prognosis.

To delineate whether SNPs are closely associated with the prognoses of patients who received adjuvant endocrine therapy alone, we again utilized the stepwise selection Cox model to analyze the SNPs identified by GWAS, and the candidate genes involved in estrogen or tamoxifen metabolisms in patients who received endocrine therapy alone, but excluded the candidate genes involved in the metabolism of chemotherapeutic agents. As shown in the Supplementary Table 2, we found that *ESR1* codon325 rs1801132 (G/G/+G/C) (involved in estrogen metabolism) was closely associated with better DDFS and *FGFR2* rs2981582 (A/A+A/G) was closely associated with poor DFS in patients who received endocrine therapy alone.

### SNPs associated with poor survival in patients with adjuvant chemotherapy

In a multiple stepwise selection Cox model, *MAP3K1* rs889312 was significantly associated with patients receiving adjuvant chemotherapy and thus poor prognosis (*P* = 0.04 for DDFS and *P* = 0.008 for DFS) (Table 2).

As shown in Supplementary Table 1 (multiple Cox model analyses adjusting conventional prognostic factors but not other SNPs), *CYP19* rs1870050 was associated with patients receiving adjuvant chemotherapy and thus poor prognosis.

## DISCUSSION

In the present study, we demonstrated that genetic variants of the host, such as SNPs of *MAP3K1*, *CYP2B6*, *UGT1A1, HCN1*, *ABCB1*, and *ALDH3A1*, may worse the prognosis of HR-positive breast cancer patients, predominantly for premenopausal women. Of them, 92.8% patients received adjuvant hormonal therapy with tamoxifen. Whether new treatment, such as the GnRh analogue plus aromatase inhibitor, improves the survival of the SNP-poor prognostic group compared to treatment with tamoxifen deserves further study. In addition, several studies have revealed that a longer duration of adjuvant hormonal therapy improves the survival of HR+ patients [19–21], and host factors may be helpful in the selection of patients who may benefit more from longer duration of hormonal therapy.

In the present study, using the multiple stepwise selection Cox model (adjusted multiple SNPs and clinicopathologic features), we found that *ESR1* codon325 rs1801132 (G/G/+G/C vs. C/C) was the only SNP significantly associated with a good DDFS, whereas *MAP3K1* and *CYP2B6* were significantly associated with poor DDFS, DFS, and OS in all women. Interestingly, *CYP2B6* rs3211371 (T/C) and *MAP3K1* rs889312 (C/C) were associated with poor prognosis in patients who receive adjuvant hormonal therapy alone, whereas *MAP3K1* rs889312 (C/C) was significantly associated with poor DDFS and DFS in patients receiving adjuvant chemotherapy. After excluding the candidate genes involved in the metabolism of chemotherapeutic agents in the multiple stepwise selection Cox model, we found that *ESR1* codon325 rs1801132 (G/G/+G/C) and *FGFR2* rs2981582 (A/A+A/G) were closely associated with better DDFS, and poor DFS, respectively, in patients who received adjuvant hormonal therapy alone (Supplementary Table 2). Although our sample size is limited (patients treated with endocrine therapy alone, n=163), these findings indicate that the variations in the genes that participate in the cell proliferation pathways (e.g. *FGFR2*) and in the metabolism of anti-hormone drugs may influence the anti-endocrine effect of the therapy, and thus determine the prognoses of this subgroup of patients. Further validation of the prognostic value of the SNPs identified in our study in a larger cohort of hormone receptor (HR)-positive patients who receive anti-hormone therapy alone is merited.

Several *CYP2B6* genotypes were associated with the metabolism of *CYP2B6* substrate drugs, including cyclophosphamide and tamoxifen, frequently used in adjuvant therapy for breast cancer [22]. In breast cancer patients receiving adjuvant chemotherapy with cyclophosphamide and doxorubicin, *CYP2B6* rs3745274 (CYP2B6*9) was reported to be associated with a poor OS [23]. Our findings showed that *CYP2B6* rs4802101 (T/T), and *CYP2B6* rs3211371 (T/C) were associated with a poor DDFS in premenopausal women. The association between certain *CYP2B6* SNPs and the outcome of breast cancer patients receiving tamoxifen alone has not yet been reported. Our study also showed that the minor allele (T) of *CYP2B6* rs3211371 was associated with poor DDF, DFS, and OS in all women, and in patients not receiving adjuvant chemotherapy supposedly with a good prognosis, but receiving tamoxifen or ovarian ablation. The C to T substitution of *CYP2B6* rs3211371 results in the substitution of arginine for cysteine; thus, it is speculated that the presence of this polymorphism may decrease the production of *CYP2B6* and further hamper the metabolism of anti-hormone agents [24–26]. However, the estimated HRs were relatively imprecise because of less frequent SNPs in the T allele.

Bochud et al. recently reported that the rare G allele of rs8099917 near the *IL28B* gene was associated with poor responses to interferon therapy in patients with chronic hepatitis C who were infected with non-1 HCV genotypes [27]. Chen et al. also described a rare germline polymorphism, *YAP1* R331W, which is associated with an increasing risk of lung adenocarcinomas [28]. Pathogenic rare variants of *BRCA2* have been found to be associated with hereditary breast and ovarian cancers by the 1000 Genomes dataset [29]. Our current study has also identified a very low minor allele frequency of 0.04 at *CYP2B6* rs3211371 (T/C), and this rare allele was found to be associated with a poor prognosis. Further exploration of this rare variant SNP, *CYP2B6* rs3211371, through a rapid growth sequencing technology and a high-density SNP genotyping array [30, 31] will enable us to have increasing opportunities to swiftly detect rare genetic alleles, and to further investigate whether these rare variants could determine the responses to treatments and the subsequent prognoses of breast cancers.

In the GWAS study, *MAP3K1* rs889312 was found to be associated with breast cancer risk [12, 32, 33]. *MAP3K1* participates in the MAPK signal transduction pathway, responding to a number of mitogenic and metabolic stimuli, including estrogen, which may influence breast cancer susceptibility by cell proliferation [32]. Growing evidence has demonstrated that MAPKs and their endogenous negative regulator, MAPK phosphatase-1 (MKP-1), may involve in the development of resistance to tamoxifen and chemotherapeutic agents [34, 35]. These mechanisms may explain why our patients with the C/C allele of *MAP3K1* rs889312 had a poor prognosis, even in patients receiving adjuvant chemotherapy.

In addition to SNPs of *CYP2B6* rs3211371 and *MAP3K1* rs889312, some SNPs of candidate genes or genes identified from GWAS were associated with poor survival, which showed (1) GWAS-identified SNP; *HCN1* rs981782, poor DDFS for all women; (2) Estrogen metabolism-associated SNP; *UGT1A1* rs4148323, poor DDFS for all women and for patients without chemotherapy; and (3) Chemotherapeutic agents for metabolism-associated SNPs; *ABCB1* rs2032582, poor OS for premenopausal women, and *ALDH3A1*, poor DFS for premenopausal women; *CYP2B6* rs4802101, and poor DDFS for premenopausal women.

In contrast to the aforementioned SNPs, SNPs of estrogen metabolism, *ESR1* codon325 rs1801132 (G/G/+G/C vs. C/C), and *ESR1 pvuII* rs2234693 (C/C+C/T vs. T/T) were associated with a better DDFS in all women, and a better OS in patients without adjuvant chemotherapy, respectively. Further study of the underlying mechanisms for the better prognosis of patients with genetic variants of *ESR1* codon325 rs1801132 and *ESR1 pvuII* rs2234693 is warranted.

Although the aforementioned SNPs did not show consistent associations between OS, DFS, and DDFS, we cannot rule out potential confounding factors resulting from the relatively small frequency of minor alleles or a proportion of local recurrence and distant metastases that were not reported but death was noted in the death registry used in this study. However, in the present study, the aforementioned SNPs were not associated with prognosis in postmenopausal women.

Previously, we had reported that *CYP19* (TTTA)n and *CYP19* genetic polymorphisms haplotype *AASA* were closely associated with poor survival in premenopausal patients with LN-negative and HR-positive breast cancers [10, 36]. In this study, we found that SNPs identified by GWAS (*MAP3K1* rs889312), and SNPs involved in the metabolism of chemotherapeutic agents (*ABCB1* rs2032582*, ALDH3A1* rs2231142, and *CYP2B6* rs4802101 and rs3211372) were associated with the prognoses in premenopausal women, but not with the prognoses in postmenopausal woman. Although we cannot rule out potential confounding effects resulting from a relatively smaller sample of postmenopausal patients, the possible reasons for the aforementioned SNPs affecting the prognoses of our premenopausal female patients are (1) the proliferation of HR-positive breast cancer cells is more estrogen-dependent in premenopausal women than in postmenopausal woman, and anti-hormone therapy (mostly with tamoxifen) or chemotherapy (partial anti-hormone effect) might cause greater decreases in the estrogen synthesized by the ovaries to support the growth of breast cancers in premenopausal women [37, 38] (2) the premenopausal women harboring the aforementioned SNPs may have higher levels of estrogen despite the anti-hormone therapy and anti-chemotherapy effects, and the existing estrogen may activate hitherto quiescent tumor cells, and may thus promote the proliferations, migrations, and distant metastases of breast cancers [36–38].

Previous studies have demonstrated that MAP3K1 could trigger the transcriptional activities of the ERs in endometrial and ovarian cancer cells [39]. In the TCGA data on breast cancers, *MAP3K1* alterations were more frequently found in the luminal A subtype than in other subtypes of breast cancers [40]. Although the relationship between estrogen levels and the SNP, *MAP3K1* rs889312, remains unclear, we speculated that the C/C allele of *MAP3K1* rs889312 may alter estrogen metabolism, and thus contribute to the progression of estrogen-dependent breast cancers, especially in premenopausal women.

In a recent study assessing the relationship between 11 GWAS-identified breast risk-associated SNPs, including *CASP8* rs17468277, *TGFB1* rs1982073, *FGFR2* rs2981582, 8q24 rs13281615, *LSP1* rs3817198, *MAP3K1* rs889312, *TOX3* rs3803662, 2q35 rs13387042, *SLC4A7* rs4973768, *COX11* rs6504950, and rs10941679 (5p12), and 62 candidate/GWAS SNPs and prognosis of 25853 breast cancer patients (with a median follow-up of 6.4 years, 15.8% died), the authors showed that only *TOX3* rs3803662 (T/T) was significantly associated with a poorer OS (HR=1.21, *P*= 0.0002, after adjusting age, tumor size, nodal status and grade) [17]. Further analyses showed that *TOX3* rs3803662 (T/T) remained a poor prognostic factor in ER-positive patients, but lost significance in ER-negative patients [17]. However, Riaz et al. showed that *TOX3* rs3803662 was not associated with a short metastasis-free survival in 1290 LN-negative breast cancer patients without adjuvant chemotherapy [41]. Our results also showed that *TOX3* (*TNRC9*) rs3803662 was not associated with the DDFS, DFS, and OS in HR-positive early breast cancer patients (71.5% are LN-negative). Another recent study evaluating 8 risk SNPs, including *FGFR2* rs1219468 and *TOX3* rs8051542, which were different from our studies of *FGFR2* rs2981582 and *TOX3* (*TNRC9*) rs3803662, showed that only two SNPs, 16q12 rs12443621 and 17q23 rs6504950, influenced OS after adjusting for age, clinical stage, and treatment [18]. The different composition of study populations may explain the different findings of our results from their studies [17, 18]. For example, our patients were HR-positive, LN node-negative, or had up to 3 positive LNs; they were also Taiwanese, had detailed information concerning their adjuvant chemotherapy regimen, and underwent long-term follow-up with a median of 10.6 years (7.2% followed for more than 15 years, 5.1% for less than 5 years). Further studies exploring the influence of GWAS-identified genes, such as 16q12 rs12443621 and 17q23 rs6504950, on the survival of HR-positive and LN node-negative breast cancers or those with up to 3 positive LNs are merited because these SNPs were reported after we genotyped our GWAS-identified genes [17, 18].

In this study, CYP2D6*10 was the only genotype associated with worse survival of postmenopausal women after adjustment for the conventional prognostic factors listed in Table 1. CYP2D6 *10 lost its significance when all the other SNPs were adjusted together in the multiple stepwise selection COX model, which may explain why the associations of *CYP2D6* and the survival of tamoxifen-treated breast cancer patients conflict in different reports. In Asians, CYP2D6*10 is the predominant polymorphism that accompanies the intermediate metabolizer phenotype, in which 2 metabolites of tamoxifen, 4-hydroxytamoxifen (4OHtam) and 4-hydroxy-N-desmethyl tamoxifen (endoxifen) exhibit greater ER affinity and are predominantly catalyzed by cytochrome *CYP2D6* [42–44]. Previous studies suggested that CYP2D6*10 alleles decreased CYP2D6 activity; thus, a shorter recurrence-free survival period was observed in Asian patients with adjuvant tamoxifen [8, 45]. Two studies reported that the poor or intermediate metabolizer of *CYP2D6* was not associated with the clinical outcome of postmenopausal Caucasian women patients with HR-positive operable invasive breast cancer receiving adjuvant tamoxifen [46, 47]. However, these studies did not include premenopausal patients and did not analyze CYP2D6*10 alleles.

In this study, 251 (60.6%) patients received different standard adjuvant chemotherapy agents, including cyclophosphamide, epirubicin, 5-fluorouracil, methotrexate, doxorubicin, and paclitaxel. In clinical practice, the choices of different standard chemotherapeutic agents and regimens made by physicians depend upon their assessments of the clinicopathological characteristics of patients, including tumor sizes, tumor grades, estrogen receptor (ER), progesterone receptor (PR), lymph nodes (LNs), underlying comorbidities in patients, and the potential toxicities of the different chemotherapy regimens. Therefore, as shown in Table 1, various chemotherapeutic agents were inevitably included in this study. However, in the current study, the standard adjuvant chemotherapy that was administered to LN-positive and LN-negative patients with high-risk factors after undergoing breast surgeries was based on the indications and the adjuvant chemotherapeutic regimens and doses described in previously published literature, or those recommended by the NCCN guidelines, the NIH consensus, and the St. Gallen consensus [2, 48, 49]. As shown in Table 4, LN-positivity, larger tumor sizes, and higher histologic grades were determining factors for patients to receive adjuvant chemotherapy. However, because only a limited number of patients in our study received adjuvant chemotherapy and heterogeneous chemotherapy regimens, the interpretations of the associations between the SNP, *MAP3K1* rs889312 (C/C), and the DDFS and DFS of patients who received adjuvant chemotherapy should be cautious. Further validation of our identified prognostic SNPs in a larger cohort of HR-positive patients with LN 1–3 who receive the same chemotherapy regimens is warranted.

**Table 4:** Multiple stepwise selection logistic regression model analyses of the predictors of patients whether receiving adjuvant chemotherapy

Covariate	aOR (95%CI)	*P*
Infiltrating ductal carcinoma + Infiltrating Lobular carcinoma+Medullary carcinoma. vs. others	23.4 (3.5-156.6)	0.001
LN 1-3 vs. 0	154.8 (19.7-999.9)	<.0001
Size	1.9 (1.4-2.7)	0.0001
Grade	1.7 (1.0-2.8)	0.03

aOR: adjusted odds ratio

In summary, our findings suggested that genetic variations in genes participating in the cell proliferation pathways and in the metabolism of anti-hormone drugs and anti-chemotherapy agents are likely to synergistically influence the outcome of HR-positive breast cancer patients. These findings provide additional evidence that the genetic variants may affect the prognosis of breast cancer. Functional analysis and validation of the biologic significances of SNPs of *CYP2B6* rs3211371 and *MAP3K1* rs889312 in this subtype of breast cancer patients are warranted. In addition, patients with *MAP3K1* rs889312 (C/C) might need different or more aggressive treatments.

## PATIENTS AND METHODS

### Study cohort and sources of information

Eligible women were newly diagnosed patients with stage I or II (AJCC 2007) HR-positive early breast cancers diagnosed at the National Taiwan University Hospital between January 1, 1994 and June 30, 2006. One pathologist (Dr. Lien) reviewed the histological grade and hormone receptor status of the primary tumor of each patient. Patients were considered HR-positive if the percentage of estrogen receptor (ER)- or progesterone receptor (PR)-positive epithelial cells was ≥ 10% [2, 50]. Genomic DNA and detailed demographic information were obtained from the patients and their medical charts with their written informed consent. The pathologic review, blood samples, and genetic studies were approved by the National Taiwan University Hospital (NTUH) ethics committee (200906075R).

Pathologic and clinical information about treatment (including type of surgery, receipt or non-receipt of adjuvant systemic therapy, and type and dose of adjuvant systemic therapy) and follow-up information (including recurrence and distant metastasis) were obtained from pathology reports and clinical records.

Patients with high-risk factors, such as grade III cancers, large tumors, and lymph node (LN) positivity (N1), all received standard adjuvant chemotherapy, such as CMF, CEF, CAF, AC/EC, or AC/EC followed by paclitaxel/docetaxel regimens as defined in our previous study [2]. In the present study, the definition of menopausal status was based on our previous study: (1) If menstruation had taken place within one year, the woman was considered to be premenopausal, and, if not, postmenopausal (2) Women who had undergone hysterectomy without bilateral oophorectomy were considered to be premenopausal if they were younger than 52 and postmenopausal if older [2, 36].

As shown in Table 4, poor prognosis factors of pathologic status, such as LN-positivity (adjusted odds ratio [aOR], 154.8; 95% CI, 19.7-999.9, *P* < 0.0001), larger tumor size (aOR, 1.9; 95% CI, 1.4-2.7, *P* = 0.0001), and higher histologic grade (aOR, 1.7; 95% CI, 1.0-2.8, *P* = 0.03) were independent factors for patients to receive adjuvant chemotherapy. All enrolled patients received adjuvant hormonal therapy. Adjuvant radiotherapy was administered to all patients after breast conservation surgery [51, 52]. After surgery and adjuvant therapy, the patients were regularly followed up in our clinic. If patients were lost to follow-up, information on disease status and survival was obtained from the patients’ charts, hospital cancer registry records, and the National Death Registry.

### Histological subgroup of HR-positive breast cancer

Histologically, tubular, mucinous, and papillary carcinomas, and ductal carcinomas *in situ* (DCIS) with microinvasions of breast cancers have more favorable prognoses than infiltrating ductal carcinomas (IDCs), infiltrating lobular carcinomas (ILCs), and medullary breast carcinomas. Unlike IDCs, the clinicopathological features of ILCs show greater association with the low-to-intermediate grade positive expression of the ER, and the negative expression or amplification of HER2 [53, 54]. However, Lorfida et al. reported that the OS of ILCs might be worse compared with those of stage-matched IDCs [55]. Although the responses to chemotherapy or treatment with aromatase inhibitors may be distinct between cases of ILCs and IDCs [56, 57], most clinical trials and practical clinical guidelines suggest that the treatments for ILCs and IDCs should be similar, and these should be considered as a single unified subtype of breast cancer. In addition, our patients with IDCs and ILCs exhibited similar 5-year DFS (81.3% versus 82.3%) and 5-year OS (87.3% versus 90.1%). Park et al. demonstrated that the prognoses of medullary breast carcinomas are not significantly different from those of IDCs, and that the prognoses were also determined by greater tumor sizes and axillary lymph node metastases [58].

As shown in Supplementary Table 3, we have demonstrated that the estimated adjusted odds ratios (aOR) for the associations of different histological subtypes with the use of adjuvant chemotherapy were more than 1 for IDCs, ILCs, or medullary carcinomas, unlike the aORs of other histological subtypes, such as mucinous carcinomas [aOR=0.5], DCIS with microinvasions, and tubular and papillary carcinomas. Although ILCs may be more endocrine-sensitive than IDCs, based on the similarities in the use of systemic chemotherapy and the prognoses, and the limited sample size of ILCs (n=16), we have included ILCs within the subgroup comprising IDCs and medullary carcinomas.

### Genotyping

TaqMan assays were used to genotype specific SNPs, including *CYP19* rs4646, rs1065779, rs1870050, and rs700519; *ESR1*, rs3020314, rs3020396, rs2982684, rs1801132, rs2234693, and rs2046210; *COMT* rs4680; *CYP3A5* rs776746; *CYP2C19* rs4244285 and rs4986893; *UGT1A1* rs4148323; *ABCB1* rs1128503, rs2032582, and rs1045642; *ALDH3A1* rs2231142 and rs2228100; *CYP2C9* rs1057910; *CYP2B6* rs4802101 and rs3211371; *FGFR2* rs2981582; *TNRC9* rs3803662; *MAP3K1* rs889312; *HCN1* rs981782, rs10941679, and rs4415084 in chromosome 5p12. The allelic frequencies of these SNPs are shown in Supplementary Table 4. The CYP2D6*10 were determined by rs16947, rs1065852, rs28371725, and rs3892097, whereas the (TTTA)n of *CYP19* were determined by performing a polymerase chain reaction (PCR) that utilized primers and methods described previously [10].

### PCR conditions for TaqMan assays

The thermal cycling conditions were 50°C for 2 minutes, 95°C for 10 minutes, followed by 40 cycles of 95°C for 15 seconds, and 60°C for 60 seconds. The PCR reaction was performed in a total reaction volume of 5 μL containing 10 ng genomic DNA, 2.5 μL of the 2X TaqMan® Universal PCR Master Mix (Applied Biosystems), and 0.125 μL of the 40X primers/probes mixed in the 384-well plate format on ABI7900HT. The primers and probes and genotyping were performed via an Assay-by-Design method or a Made to Order Assay (Applied Biosystems).

### Statistical analysis

Follow-up data available as of December 31, 2011 were analyzed. Distant disease-free survival (DDFS) was measured from the date of the original surgery for breast cancer to distant recurrence or death from any cause, disease-free survival (DFS) was measured from the date of the original surgery for breast cancer to local recurrence, distant recurrence or death from any cause and overall survival (OS) was measured from the date of the original surgery to the date of death from any cause or the last follow-up date [52]. Multiple-adjusted hazard ratio (HR) (aHR) of disease status associated with the individual genotype was assessed after adjustment for age, menopausal status, tumor size, grade, ER, PR, LN status, histopathology, adjuvant chemotherapy, and adjuvant hormonal therapy in the multiple Cox model (data are shown in Supplementary Table 1).

Furthermore, all SNPs and all the above mentioned variables were further analyzed by the stepwise variable selection procedure with the significance level for entry (SLE) and the significance level for stay (SLS) set to 0.05 (data shown in Table 2). The stepwise selection Cox model was used to identify the variables that showed significant associations with disease status. In the subgroup analysis, including premenopausal patients, postmenopausal patients, patients receiving adjuvant chemotherapy, and patients receiving adjuvant hormonal therapy alone, stepwise selection was continued as conducted in subgroup analysis.

The stepwise selection Cox model has been widely used to predict the hazard rates in patients in various clinical epidemiological studies, such as, those conducted by Yang et al. [59], and Pande et al. [60]. Stepwise regression is a combination of the forward and backward selection techniques. During the iterative process of variable selection, variables are removed from the model if they are deemed non-significant. Furthermore, the whole stepwise procedure repeats between the forward and backward steps until no additional variables are added to the current model. Therefore, in our study, after the stepwise selection procedures were completed, sets of significant variables were selected and listed in Table 2. All analyses were performed using SAS statistical software for Windows version 9.2 (SAS Institute, Cary, NC, USA).

## SUPPLEMENTARY MATERIALS FIGURES AND TABLES





## References

[1] Breast Early (2005). Cancer Trialists’ Collaborative Group (EBCTCG). Effects of chemotherapy and hormonal therapy for early breast cancer on recurrence and 15-year survival: an overview of the randomised trials. Lancet.

[2] Kuo SH, Lien HC, You SL, Lu YS, Lin CH, Chen TZ, Huang CS (2008). Dose variation and regimen modification of adjuvant chemotherapy in daily practice affect survival of stage I-II and operable stage III Taiwanese breast cancer patients. Breast.

[3] Sørlie T, Perou CM, Tibshirani R, Aas T, Geisler S, Johnsen H, Hastie T, Eisen MB, van de Rijn M, Jeffrey SS, Thorsen T, Quist H, Matese JC (2001). Gene expression patterns of breast carcinomas distinguish tumor subclasses with clinical implications. Proc Natl Acad Sci USA.

[4] Paik S, Shak S, Tang G, Kim C, Baker J, Cronin M, Baehner FL, Walker MG, Watson D, Park T, Hiller W, Fisher ER, Wickerham DL (2004). A multigene assay to predict recurrence of tamoxifen-treated, node-negative breast cancer. N Engl J Med.

[5] Sotiriou C, Pusztai L (2009). Gene-expression signatures in breast cancer. N Engl J Med.

[6] Sorlie T (2007). Molecular classification of breast tumors: toward improved diagnostics and treatments. Methods Mol Biol.

[7] Huang CS, Chern HD, Chang KJ, Cheng CW, Hsu SM, Shen CY (1999). Breast cancer risk associated with genotype polymorphism of the estrogen-metabolizing genes CYP17, CYP1A1, and COMT: a multigenic study on cancer susceptibility. Cancer Res.

[8] Nowell S, Sweeney C, Winters M, Stone A, Lang NP, Hutchins LF, Kadlubar FF, Ambrosone CB (2002). Association between sulfotransferase 1A1 genotype and survival of breast cancer patients receiving tamoxifen therapy. J Natl Cancer Inst.

[9] Kiyotani K, Mushiroda T, Sasa M, Bando Y, Sumitomo I, Hosono N, Kubo M, Nakamura Y, Zembutsu H (2008). Impact of CYP2D6*10 on recurrence-free survival in breast cancer patients receiving adjuvant tamoxifen therapy. Cancer Sci.

[10] Huang CS, Kuo SH, Lien HC, Yang SY, You SL, Shen CY, Lin CH, Lu YS, Chang KJ (2008). The CYP19 TTTA repeat polymorphism is related to the prognosis of premenopausal stage I-II and operable stage III breast cancers. Oncologist.

[11] Huang CS, Lin CH, Lu YS, Shen CY (2010). Unique features of breast cancer in Asian women--breast cancer in Taiwan as an example. J Steroid Biochem Mol Biol.

[12] Easton DF, Pooley KA, Dunning AM, Pharoah PD, Thompson D, Ballinger DG, Struewing JP, Morrison J, Field H, Luben R, Wareham N, Ahmed S, Healey CS (2007). Genome-wide association study identifies novel breast cancer susceptibility loci. Nature.

[13] Hunter DJ, Kraft P, Jacobs KB, Cox DG, Yeager M, Hankinson SE, Wacholder S, Wang Z, Welch R, Hutchinson A, Wang J, Yu K, Chatterjee N (2007). A genome-wide association study identifies alleles in FGFR2 associated with risk of sporadic postmenopausal breast cancer. Nat Genet.

[14] Nordgard SH, Johansen FE, Alnaes GI, Naume B, Børresen-Dale AL, Kristensen VN (2007). Genes harbouring susceptibility SNPs are differentially expressed in the breast cancer subtypes. Breast Cancer Res.

[15] Kristensen VN, Sørlie T, Geisler J, Langerød A, Yoshimura N, Kåresen R, Harada N, Lønning PE, Børresen-Dale AL (2005). Gene expression profiling of breast cancer in relation to estrogen receptor status and estrogenmetabolizing enzymes: clinical implications. Clin Cancer Res.

[16] Stacey SN, Manolescu A, Sulem P, Thorlacius S, Gudjonsson SA, Jonsson GF, Jakobsdottir M, Bergthorsson JT, Gudmundsson J, Aben KK, Strobbe LJ, Swinkels DW, van Engelenburg KC (2008). Common variants on chromosome 5p12 confer susceptibility to estrogen receptor-positive breast cancer. Nat Genet.

[17] Fasching PA, Pharoah PD, Cox A, Nevanlinna H, Bojesen SE, Karn T, Broeks A, van Leeuwen FE, van’t Veer LJ, Udo R, Dunning AM, Greco D, Aittomäki K (2012). The role of genetic breast cancer susceptibility variants as prognostic factors. Hum Mol Genet.

[18] Bayraktar S, Thompson PA, Yoo SY, Do KA, Sahin AA, Arun BK, Bondy ML, Brewster AM (2013). The relationship between eight GWAS-identified single-nucleotide polymorphisms and primary breast cancer outcomes. Oncologist.

[19] Pater J, Tu D, Shepherd L, Ingle JN, Goss PE (2008). Decision making in adjuvant trials in breast cancer: the NCIC CTG MA.17 trial as an example. Breast Cancer Res Treat.

[20] Davies C, Pan H, Godwin J, Ingle JN, Goss PE (2013). Adjuvant Tamoxifen: Longer Against Shorter (ATLAS) Collaborative Group. Long-term effects of continuing adjuvant tamoxifen to 10 years versus stopping at 5 years after diagnosis of oestrogen receptor-positive breast cancer: ATLAS, a randomised trial. Lancet.

[21] Schiavon G, Smith IE (2014). Status of adjuvant endocrine therapy for breast cancer. Breast Cancer Res.

[22] Wang H, Tompkins LM (2008). CYP2B6: new insights into a historically overlooked cytochrome P450 isozyme. Curr Drug Metab.

[23] Bray J, Sludden J, Griffin MJ, Cole M, Verrill M, Jamieson D, Boddy AV (2010). Influence of pharmacogenetics on response and toxicity in breast cancer patients treated with doxorubicin and cyclophosphamide. Br J Cancer.

[24] Lo R, Burgoon L, Macpherson L, Ahmed S, Matthews J (2010). Estrogen receptor-dependent regulation of CYP2B6 in human breast cancer cells. Biochim Biophys Acta.

[25] Lang T, Klein K, Fischer J, Nüssler AK, Neuhaus P, Hofmann U, Eichelbaum M, Schwab M, Zanger UM (2001). Extensive genetic polymorphism in the human CYP2B6 gene with impact on expression and function in human liver. Pharmacogenetics.

[26] Justenhoven C, Pentimalli D, Rabstein S, Harth V, Lotz A, Pesch B, Brüning T, Dörk T, Schürmann P, Bogdanova N, Park-Simon TW, Couch FJ, Olson JE (2014). CYP2B6*6 is associated with increased breast cancer risk. Int J Cancer.

[27] Bochud PY, Bibert S, Kutalik Z, Patin E, Guergnon J, Nalpas B, Goossens N, Kuske L, Müllhaupt B, Gerlach T, Heim MH, Moradpour D, Cerny A (2012). IL-28B alleles associated with poor hepatitis C virus (HCV) clearance protect against inflammation and fibrosis in patients infected with non-1 HCV genotypes. Hepatology.

[28] Chen HY, Yu SL, Ho BC, Su KY, Hsu YC, Chang CS, Li YC, Yang SY, Hsu PY, Ho H, Chang YH, Chen CY, Yang HI (2015). R331W missense mutation of oncogene YAP1 is a germline risk allele for lung adenocarcinoma with medical actionability. J Clin Oncol.

[29] Olfson E, Cottrell CE, Davidson NO, Gurnett CA, Heusel JW, Stitziel NO, Chen LS, Hartz S, Nagarajan R, Saccone NL, Bierut LJ (2015). Identification of Medically Actionable Secondary Findings in the 1000 Genomes. PLOS one.

[30] Chen CY, Chang IS, Hsiung CA, Wasserman WW (2014). On the identification of potential regulatory variants within genome wide association candidate SNPsets. BMC Med Genomics.

[31] Lin PH, Kuo WH, Huang AC, Lu YS, Lin CH, Kuo SH, Wang MY, Liu CY, Cheng FT, Yeh MH, Li HY, Yang YH, Hsu YH (2016). Multiple gene sequencing for risk assessment in patients with early-onset or familial breast cancer. Oncotarget.

[32] Klinge CM, Blankenship KA, Risinger KE, Bhatnagar S, Noisin EL, Sumanasekera WK, Zhao L, Brey DM, Keynton RS (2005). Resveratrol and estradiol rapidly activate MAPK signaling through estrogen receptors alpha and beta in endothelial cells. J Biol Chem.

[33] Lu PH, Yang J, Li C, Wei MX, Shen W, Shi LP, Jiang ZY, Zhou N, Tao GQ (2011). Association between mitogen-activated protein kinase kinase kinase 1 rs889312 polymorphism and breast cancer risk: evidence from 59,977 subjects. Breast Cancer Res Treat.

[34] Small GW, Shi YY, Higgins LS, Orlowski RZ (2007). Mitogen-activated protein kinase phosphatase-1 is a mediator of breast cancer chemoresistance. Cancer Res.

[35] Haagenson KK, Wu GS (2010). The role of MAP kinases and MAP kinase phosphatase-1 in resistance to breast cancer treatment. Cancer Metastasis Rev.

[36] Kuo SH, Yang SY, Lien HC, Lo C, Lin CH, Lu YS, Cheng AL, Chang KJ, Huang CS (2013). CYP19 genetic polymorphism haplotype AASA is associated with a poor prognosis in premenopausal women with lymph node-negative, hormone receptor-positive breast cancer. BioMed Research International.

[37] Santen RJ, Brodie H, Simpson ER, Siiteri PK, Brodie A (2009). History of aromatase: saga of an important biological mediator and therapeutic target. Endocr Rev.

[38] Clarke R, Tyson JJ, Dixon JM (2015). Endocrine resistance in breast cancer--An overview and update. Mol Cell Endocrinol.

[39] Lee H, Jiang F, Wang Q, Nicosia SV, Yang J, Su B, Bai W (2000). MEKK1 activation of human estrogen receptor alpha and stimulation of the agonistic activity of 4-hydroxytamoxifen in endometrial and ovarian cancer cells. Mol Endocrinol.

[40] Cerami E, Gao J, Dogrusoz U, Gross BE, Sumer SO, Aksoy BA, Jacobsen A, Byrne CJ, Heuer ML, Larsson E, Antipin Y, Reva B, Goldberg AP (2012). The cBio cancer genomics portal: an open platform for exploring multidimensional cancer genomics data. Cancer Discov.

[41] Riaz M, Berns EM, Sieuwerts AM, Ruigrok-Ritstier K, de Weerd V, Groenewoud A, Uitterlinden AG, Look MP, Klijn JG, Sleijfer S, Foekens JA, Martens JW (2012). Correlation of breast cancer susceptibility loci with patient characteristics, metastasis-free survival, and mRNA expression of the nearest genes. Breast Cancer Res Treat.

[42] Pasqualini JR, Sumida C, Giambiagi N (1988). Pharmacodynamic and biological effects of anti-estrogens in different models. J Steroid Biochem.

[43] Jin Y, Desta Z, Stearns V, Ward B, Ho H, Lee KH, Skaar T, Storniolo AM, Li L, Araba A, Blanchard R, Nguyen A, Ullmer L (2005). CYP2D6 genotype, antidepressant use, andtamoxifen metabolism during adjuvant breast cancer treatment. J Natl Cancer Inst.

[44] Rodriguez-Antona C, Ingelman-Sundberg M (2006). Cytochrome P450 pharmacogenetics and cancer. Oncogene.

[45] Xu Y, Sun Y, Yao L, Shi L, Wu Y, Ouyang T, Li J, Wang T, Fan Z, Fan T, Lin B, He L, Li P (2008). Association between CYP2D6*10 genotype and survival of breast cancer patients receiving tamoxifen treatment. Ann Oncol.

[46] Rae JM, Drury S, Hayes DF, Stearns V, Thibert JN, Haynes BP, Salter J, Sestak I, Cuzick J, M; Dowsett (2012). ATAC trialists. CYP2D6 and UGT2B7 genotype and risk of recurrence in tamoxifen-treated breast cancer patients. J Natl Cancer Inst.

[47] Regan MM, Leyland-Jones B, Bouzyk M, Pagani O, Tang W, Kammler R, Dell’orto P, Biasi MO, Thürlimann B, Lyng MB, Ditzel HJ, Neven P, Debled M (2012). CYP2D6 genotype and tamoxifen response in postmenopausal women with endocrine-responsive breast cancer: the breast international group 1-98 trial. J Natl Cancer Inst.

[48] Goldhirsch A, Ingle JN, Gelber RD, Coates AS, Thurlimann B, Senn HJ (2009). Thresholds for therapies: highlights of the St Gallen International Expert Consensus on the primary therapy of early breast cancer 2009. Ann Oncol.

[49] Lu YS, Kuo SH, Huang CS (2004). Recent advances in the management of primary breast cancers. J Formos Med Assoc.

[50] Lin CH, Lien HC, Hu FC, Lu YS, Kuo SH, Wu LC, You SL, Cheng AL, Chang KJ, Huang CS (2010). Fractionated evaluation of immunohistochemical hormone receptor expression enhances prognostic prediction in breast cancer patients treated with tamoxifen as adjuvant therapy. J Zhejiang Univ Sci B.

[51] Kaufmann M, Morrow M, von Minckwitz G, Harris JR (2010). Locoregional treatment of primary breast cancer: consensus recommendations from an International Expert Panel. Cancer.

[52] Hudis CA, Barlow WE, Costantino JP, Gray RJ, Pritchard KI, Chapman JA, Sparano JA, Hunsberger S, Enos RA, Gelber RD, Zujewski JA (2007). Proposal for standardized definitions for efficacy end points in adjuvant breast cancer trials: the STEEP system. J Clin Oncol.

[53] Rakha EA, Ellis IO Lobular breast carcinoma and its variants. Semin Diagn Pathol.

[54] Christgen M, Steinemann D, Kühnle E, Länger F, Gluz O, Harbeck N, Kreipe H Lobular breast cancer: Clinical, molecular and morphological characteristics. Pathol Res Pract.

[55] Iorfida M, Maiorano E, Orvieto E, Maisonneuve P, Bottiglieri L, Rotmensz N, Montagna E, Dellapasqua S, Veronesi P, Galimberti V, Luini A, Goldhirsch A, Colleoni M Invasive lobular breast cancer: subtypes and outcome. Breast Cancer Res Treat.

[56] Rakha EA, El-Sayed ME, Powe DG, Green AR, Habashy H, Grainge MJ, Robertson JF, Blamey R, Gee J, Nicholson RI, Lee AH, Ellis IO Invasive lobular carcinoma of the breast: response to hormonal therapy and outcomes. Eur J Cancer.

[57] Barroso-Sousa R, Metzger-Filho O Differences between invasive lobular and invasive ductal carcinoma of the breast: results and therapeutic implications. Ther Adv Med Oncol.

[58] Park I, Kim J, Kim M, Bae SY, Lee SK, Kil WH, Lee JE, Nam SJ (2013). Comparison of the characteristics of medullary breast carcinoma and invasive ductal carcinoma. J Breast Cancer.

[59] Yang CH, Yu CJ, Shih JY, Chang YC, Hu FC, Tsai MC, Chen KY, Lin ZZ, Huang CJ, Shun CT, Huang CL, Bean J, Cheng AL (2008). Specific EGFR mutations predict treatment outcome of stage IIIB/IV patients with chemotherapy-naive non-small-cell lung cancer receiving first-line gefitinib monotherapy. J Clin Oncol.

[60] Pande M, Spitz MR, Wu X, Gorlov IP, Chen WV, Amos CI (2011). Novel genetic variants in the chromosome 5 p15.33 region associate with lung cancer risk. Carcinogenesis.

