# Supplementary material for: Polymorphisms of ESR1, UGT1A1, HCN1, MAP3K1 and CYP2B6 are associated with the prognosis of hormone receptor-positive early breast cancer
Source: Oncotarget. 2017 Feb 2;8(13):20925–38. doi: 10.18632/oncotarget.14995 (PMC5400556; doi:10.18632/oncotarget.14995)
Supplement: Supplementary file 1 [file oncotarget-08-20925-s001.pdf]

Polymorphisms of *ESR1*, *UGT1A1*, *HCN1*, *MAP3K1* and *CYP2B6* are associated with the prognosis of hormone receptor-positive early breast cancer

SUPPLEMENTARY TABLES

Supplementary Table 1: The associations of hormone receptor-positive breast cancer patients with various genotypes and stratification according to menopausal status or adjuvant hormone therapy or chemotherapy in multiple cox model.

See Supplementary File 1

Supplementary Table 2: Multiple stepwise selection cox model of the predictors of survival in hormone receptor-positive early breast cancer patients receiving adjuvant hormonal therapy alone (number=163)

SNP	DDFS		DFS		OS	
	aHR (95%CI)	*P*	aHR (95%CI)	*P*	aHR (95%CI)	*P*
ESR1 codon325 rs1801132 (G/G/+G/C vs. C/C)	0.4 (0.2-0.9)	0.02				
FGFR2 rs2981582 (A/A+A/G vs. G/G)			2.2 (1.0-4.6)	0.05		

Abbreviation: SNP, single nucleotide polymorphisms; DDFS, distant disease-free survival; DFS, disease-free survival; OS, overall survival; aHR, adjusted hazard ratios.

Supplementary Table 3: Multiple logistic regression model analyses of the predictors of patients whether receiving adjuvant chemotherapy

Covariate	aOR (95%CI)	*P*
Infiltrating ductal carcinoma vs. Other histological subtypes (DCIS and microinvasions, tubular and papillary cancer)	18.7 (2.5-138.3)	0.004
Infiltrating Lobular ca. vs. other histological subtypes (DCIS and microinvasions, tubular and papillary cancer)	2.5 (0.2-32.1)	0.48
Medullary ca. vs. others histological subtype (DCIS and microinvasions, tubular and papillary cancer)	135 (0.6-328.8)	0.11
Mucinous ca. vs. others (DCIS and microinvasions, tubular and papillary cancer)	0.5 (0.02-11.3)	0.64

Abbreviations: aOR (95% CI), adjustment by age, menopausal status, tumor size, grade, estrogen receptor, progesterone receptor, lymph nodes status, and histopathology; Ca, carcinoma; DCIS, ductal carcinoma in situ.

Supplementary Table 4: Allelic frequency of screened SNPs

Marker	Allelic frequency
CYP19 rs4646 C/A	0.70/0.30
CYP19 rs1065779 C/A	0.53/0.47
CYP19 rs1870050 A/C	0.77/0.23
CYP19 rs700519 C/T	0.85/0.15
ESR1_intron4 rs3020314 C/T	0.66/0.34
ESR1_intron4 rs3020396 G/A	0.64/0.36
ESR1_intron4 rs2982684 C/A	0.56/0.44
ESR1_325 rs1801132 G/C	0.52/0.48
ESR1_pvuII rs2234693 T/C	0.63/0.37
ESR1 rs2046210 G/A	0.59/0.41
COMT rs4680 G/T	0.72/0.28
CYP3A5 rs776746 C/T	0.73/0.27
CYP2C19 rs4244285 G/A	0.70/0.30
CYP2C19 rs4986893 G /A	0.94/0.06
UGT1A1 rs4148323 G/A	0.83/0.17
ABCB1_Gln rs1128503 A/G	0.69/0.31
ABCB1 rs2032582 C/T	0.80/0.20
ABCB1 rs1045642 G/A	0.63/0.37
ALDH3A1 rs2231142 G/T	0.68/0.32
ALDH3A1 rs2228100 G/ C	0.55/0.45
CYP2C9 rs1057910 A/C	0.98/0.02
CYP2B6 rs4802101 C/T	0.71/0.29
CYP2B6 rs3211371 C/T	0.96/0.04
FGFR2 rs2981582 G/A	0.67/0.33
TNRC9 rs3803662 A/G	0.65/0.35
MAP3K1 rs889312 C/A	0.50/0.50
HCN1 rs981782 A/C	0.67/0.33
5p12 rs10941679 A/G	0.57/0.43
5p12 rs4415084 C/T	0.50/0.50
