# Supplementary material for: Polymorphisms of ESR1, UGT1A1, HCN1, MAP3K1 and CYP2B6 are associated with the prognosis of hormone receptor-positive early breast cancer
Source: Oncotarget. 2017 Feb 2;8(13):20925–38. doi: 10.18632/oncotarget.14995 (PMC5400556; doi:10.18632/oncotarget.14995)
Supplement: Supplementary file 2 [file oncotarget-08-20925-s002.doc]

**Supplementary Table 1. The Associations of Hormone Receptor-Positive Breast Cancer Patients with Various Genotypes and Stratification According to Menopausal Status or Adjuvant Hormone Therapy or Chemotherapy in multiple Cox model**

|  | **DDFS** | | **DFS** |  | | | **OS** | | | | | |  | |
| --- | --- | --- | --- | --- | --- | --- | --- | --- | --- | --- | --- | --- | --- | --- |
| **Genotype** | **aHR (95%CI)** | ***P*** | **aHR (95%CI)** | ***P*** | | | **aHR (95%CI)** | | | ***P*** | | |  | |
| **CYP19_(TTTA)n (S/S + S/L vs. L/L)** | | |  |  | | |  | | |  | | |  | |
| Total  (N=414) | 1.3 (0.7-2.4) | 0.44 | 1.5 (0.9-2.7) | 0.15 | | | 1.4 (0.7-2.9) | | | 0.38 | | |  | |
| Premenopausal  (n=251) | 1.7 (0.8-3.7) | 0.18 | 1.9 (0.9-3.5) | 0.07 | | | 2.0 (0.7-5.3) | | | 0.13 | | |  | |
| Postmenopausal  (n=163) | 0.9 (0.3-2.4) | 0.78 | 1.0 (0.4-2.7) | 0.99 | | | 0.7 (0.2-2.4) | | | 0.63 | | |  | |
| Receiving adjuvant chemotherapy (n=251) | 0.8 (0.4-1.6) | 0.53 | 0.9 (0.5-1.8) | 0.83 | | | 0.8 (0.4-1.9) | | | 0.64 | | |  | |
| Without receiving adjuvant chemotherapy (n=163) | 7.1 (0.9-53.4) | 0.06 | 6.6 (1.5-28.7) | 0.01 | | | 6.7(0.9-51.6) | | | 0.07 | | |  | |
| **CYP19_rs4646 (A/A +A/C vs. C/C)** | | |  |  | | |  | | |  | | |  | |
| Total  (N=414) | 1.5 (0.9-2.4) | 0.09 | 1.5 (0.9-2.2) | 0.07 | | | 1.7(0.9-2.9) | | | 0.08 | | |  | |
| Premenopausal  (n=251) | 2.0 (1.1-3.6) | 0.02 | 1.5 (0.9-2.5) | 0.11 | | | 2.0 (0.9-4.2) | | | 0.07 | | |  | |
| Postmenopausal  (n=163) | 1.1 (0.5-2.6) | 0.77 | 1.5 (0.7-3.3) | 0.27 | | | 1.3 (0.5-3.2) | | | 0.61 | | |  | |
| Receiving adjuvant chemotherapy (n=251) | 1.5 (0.8-2.7) | 0.20 | 1.4 (0.8-2.4) | 0.26 | | | 1.5 (0.7-3.3) | | | 0.25 | | |  | |
| Without receiving adjuvant chemotherapy (n=163) | 1.8 (0.8-4.0) | 0.16 | 1.7 (0.9-3.4) | 0.12 | | | 2.0 (0.8-4.8) | | | 0.14 | | |  | |
| **CYP19_rs1870050 (A/A vs. C/C +A/C)** | | |  |  | | |  | | |  | | |  | |
| Total  (N=414) | 1.8 (1.1-3.0) | 0.02 | 1.5 (1.0-2.3) | 0.07 | | | 1.5 (0.8-2.6) | | | 0.19 | | |  | |
| Premenopausal  (n=251) | 2.1 (1.1-4.0) | 0.02 | 1.6 (0.9-2.8) | 0.07 | | | 2.0 (0.9-4.3) | | | 0.08 | | |  | |
| Postmenopausal  (n=163) | 1.5 (0.6-3.3) | 0.36 | 1.2 (0.6-2.5) | 0.65 | | | 1.0 (0.4-2.5) | | | 0.98 | | |  | |
| Receiving adjuvant chemotherapy (n=251) | 1.9 (1.0-3.7) | 0.05 | 1.5 (0.8-2.6) | 0.19 | | | 1.4 (0.6-2.9) | | | 0.46 | | |  | |
| Without receiving adjuvant chemotherapy (n=163) | 1.8 (0.8-3.9) | 0.14 | 1.8 (0.9-3.4) | 0.10 | | | 1.7 (0.7-4.2) | | | 0.22 | | |  | |
| **CYP19_rs700519 (T/T+T/C vs. C/C)** | | |  |  | | |  | | |  | | |  | |
| Total  (N=414) | 1.7 (0.9-3.1) | 0.08 | 1.5 (0.9-2.5) | 0.15 | | | 2.7 (1.2-6.4) | | | 0.02 | | |  | |
| Premenopausal  (n=251) | 1.5 (0.7-3.0) | 0.26 | 1.1 (0.6-2.0) | 0.71 | | | 2.0 (0.8-5.2) | | | 0.17 | | |  | |
| Postmenopausal  (n=163) | 2.2 (0.7-7.5) | 0.20 | 2.9 (0.9-9.5) | 0.09 | | | 6.1 (0.8-46.2) | | | 0.08 | | |  | |
| Receiving adjuvant chemotherapy (n=251) | 1.9 (0.9-4.0) | 0.12 | 1.6 (0.8-3.2) | 0.20 | | | 2.6 (0.9-7.5) | | | 0.08 | | |  | |
| Without receiving adjuvant chemotherapy (n=163) | 1.9 (0.6-5.4) | 0.26 | 1.2 (0.5-6.8) | 0.67 | | | 3.5 (0.8-15.5) | | | 0.09 | | |  | |
| **CYP19_rs1065779 (A/A+A/C vs. C/C)** | | |  |  | | |  | | |  | | |  | |
| Total  (N=414) | 1.2 (0.7-1.9) | 0.56 | 1.3 (0.8-2.1) | 0.23 | | | 1.1 (0.6-2.0) | | | 0.75 | | |  | |
| Premenopausal  (n=251) | 1.5 (0.8-2.9) | 0.23 | 1.6 (0.9-2.8) | 0.13 | | | 1.5 (0.7-3.3) | | | 0.34 | | |  | |
| Postmenopausal  (n=163) | 0.9 (0.3-1.9) | 0.62 | 1.0 (0.4-2.3) | 0.98 | | | 0.6 (0.2-1.6) | | | 0.36 | | |  | |
| Receiving adjuvant chemotherapy (n=251) | 1.0 (0.5-2.0) | 0.90 | 1.1 (0.6-1.9) | 0.85 | | | 0.9 (0.4-2.1) | | | 0.89 | | |  | |
| Without receiving adjuvant chemotherapy (n=163) | 1.4 (0.6-3.3) | 0.48 | 2.1 (0.9-4.7) | 0.08 | | | 1.4 (0.5-3.6) | | | 0.53 | | |  | |
| **ESR1_intron4 rs3020314 (T/T+T/C vs. C/C)** | | |  |  | | |  | | |  | | |  | |
| Total  (N=414) | 1.3 (0.8-2.1) | 0.33 | 1.0 (0.7-1.6) | 0.88 | | | 1.2 (0.7-2.1) | | | 0.53 | | |  | |
| Premenopausal  (n=251) | 1.1 (0.6-2.1) | 0.69 | 1.0 (0.6-1.7) | 0.93 | | | 1.1 (0.5-2.4) | | | 0.77 | | |  | |
| Postmenopausal  (n=163) | 1.5 (0.7-3.4) | 0.34 | 1.1 (0.5-2.4) | 0.83 | | | 1.1 (0.5-2.8) | | | 0.79 | | |  | |
| Receiving adjuvant chemotherapy (n=251) | 1.4 (0.7-2.5) | 0.35 | 1.2 (0.7-2.1) | 0.54 | | | 1.4 (0.7-3.1) | | | 0.34 | | |  | |
| Without receiving adjuvant chemotherapy (n=163) | 1.3 (0.6-2.8) | 0.57 | 0.9 (0.4-1.8) | 0.74 | | | 0.9 (0.4-2.4) | | | 0.88 | | |  | |
| **ESR1_intron4 rs3020396 (G/G+GA vs. A/A)** | | |  |  | | |  | | |  | | |  | |
| Total  (N=414) | 0.9 (0.4-2.0) | 0.86 | 0.7 (0.4-1.3) | 0.30 | | | 0.8 (0.3-1.8) | | | 0.57 | | |  | |
| Premenopausal  (n=251) | 0.8 (0.4-1.8) | 0.60 | 0.7 (0.3-1.3) | 0.21 | | | 0.7 (0.3-1.8) | | | 0.44 | | |  | |
| Postmenopausal  (n=163) | 1.8 (0.2-14.4) | 0.59 | 1.2 (0.3-5.3) | 0.84 | | | 1.3 (0.2-10.6) | | | 0.79 | | |  | |
| Receiving adjuvant chemotherapy (n=251) | 0.6 (0.3-1.2) | 0.14 | 0.5 (0.3-1.1) | 0.09 | | | 0.5 (0.2-1.2) | | | 0.11 | | |  | |
| Without receiving adjuvant chemotherapy (n=163) | - | - | 1.3 (0.4-4.4) | 0.70 | | | - | | | -- | | |  | |
| **ESR1_intron4_rs2982684 (C/C+C/A vs. A/A)** | | |  |  | | |  | | |  | | |  | |
| Total  (N=414) | 1.0 (0.7-1.8) | 0.99 | 1.2 (0.7-1.9) | 0.60 | | | 1.2 (0.6-2.3) | | | 0.69 | | |  | |
| Premenopausal  (n=251) | 1.2 (0.6-2.6) | 0.54 | 1.3 (0.7-2.5) | 0.37 | | | 1.5 (0.6-3.8) | | | 0.35 | | |  | |
| Postmenopausal  (n=163) | 0.7 (0.2-1.8) | 0.42 | 0.7 (0.3-1.9) | 0.55 | | | 0.7 (0.2-2.2) | | | 0.52 | | |  | |
| Receiving adjuvant chemotherapy (n=251) | 0.8 (0.4-1.7) | 0.60 | 1.1 (0.5-2.2) | 0.85 | | | 1.2 (0.5-3.3) | | | 0.66 | | |  | |
| Without receiving adjuvant chemotherapy (n=163) | 1.2 (0.5-3.2) | 0.67 | 1.2 (0.5-2.7) | 0.63 | | | 1.1 (0.4-3.0) | | | 0.87 | | |  | |
| **ESR1_325 rs1801132 (G/G/+G/C vs. C/C)** | | |  | |  | | |  | | |  | | | |
| Total  (N=414) | 0.6 (0.4-1.1) | 0.08 | 0.8 (0.5-1.2) | 0.24 | | | 0.8 (0.4-1.5) | | | 0.48 | | |  | |
| Premenopausal  (n=251) | 0.6 (0.3-1.1) | 0.12 | 0.8 (0.4-1.4) | 0.39 | | | 0.7 (0.3-1.5) | | | 0.37 | | |  | |
| Postmenopausal  (n=163) | 0.7 (0.3-1.7) | 0.39 | 0.7 (0.3-1.7) | 0.48 | | | 1.2 (0.4-3.8) | | | 0.76 | | |  | |
| Receiving adjuvant chemotherapy (n=251) | 0.8 (0.4-1.7) | 0.59 | 1.0 (0.5-1.9) | 0.93 | | | 1.0 (0.4-2.6) | | | 0.93 | | |  | |
| Without receiving adjuvant chemotherapy (n=163) | 0.4 (0.2-0.9) | 0.03 | 0.5 (0.3-1.1) | 0.08 | | | 0.5 (0.2-1.4) | | | 0.22 | | |  | |
| **ESR1_pvuII rs2234693 (C/C+C/T vs.T/T)** | | |  |  | | |  | | |  | | |  | |
| Total  (N=414) | 1.1 (0.7-1.7) | 0.83 | 1.0 (0.7-1.5) | 0.98 | | | 0.9 (0.5-1.6) | | | 0.75 | | |  | |
| Premenopausal  (n=251) | 1.0 (0.5-1.7) | 0.91 | 0.9 (0.5-1.5) | 0.72 | | | 0.9 (0.4-1.9) | | | 0.79 | | |  | |
| Postmenopausal  (n=163) | 1.2 (0.5-2.9) | 0.69 | 1.2 (0.5-2.8) | 0.62 | | | 0.8 (0.3-2.2) | | | 0.69 | | |  | |
| Receiving adjuvant chemotherapy (n=251) | 1.3 (0.7-2.5) | 0.36 | 1.3 (0.7-2.3) | 0.35 | | | 1.5 (0.7-3.4) | | | 0.29 | | |  | |
| Without receiving adjuvant chemotherapy (n=163) | 0.7 (0.3-1.5) | 0.38 | 0.7 (0.3-1.3) | 0.23 | | | 0.4 (0.2-1.0) | | | 0.06 | | |  | |
| **ESR1 rs2046210 (A/A vs. A/G+G/G)** | | |  |  | | |  | | |  | | |  | |
| Total  (N=414) | 0.7 (0.3-1.4) | 0.29 | 0.6 (0.3-1.2) | 0.18 | | | 0.8 (0.4-1.9) | | | 0.66 | | |  | |
| Premenopausal  (n=251) | 1.0 (0.4-2.1) | 0.91 | 0.9 (0.5-1.9) | 0.85 | | | 1.3 (0.5-3.5) | | | 0.55 | | |  | |
| Postmenopausal  (n=163) | 0.2(0.03-1.5) | 0.12 | 0.2 (0.02-1.1) | 0.07 | | | 0.3 (0.03-2.0) | | | 0.19 | | |  | |
| Receiving adjuvant chemotherapy (n=251) | 0.7 (0.3-1.5) | 0.33 | 0.6 (0.3-1.4) | 0.26 | | | 0.8 (0.3-2.1) | | | 0.59 | | |  | |
| Without receiving adjuvant chemotherapy (n=163) | 0.5 (0.1-2.3) | 0.40 | 0.7 (0.2-2.4) | 0.56 | | | 1.0 (0.2-4.7) | | | 0.98 | | |  | |
| **COMT rs4680 ( A/A vs. A/G+G/G)** | | |  | |  | | |  | | |  | | |  |
| Total  (N=414) | 1.6 (0.7-3.7) | 0.23 | 1.2 (0.6-2.7) | 0.61 | | | 2.2 (0.9-5.6) | | | 0.08 | | |  | |
| Premenopausal  (n=251) | 2.4 (0.8-6.9) | 0.11 | 1.4 (0.5-4.0) | 0.54 | | | 4.6 (1.5-13.8) | | | 0.007 | | |  | |
| Postmenopausal  (n=163) | 1.1 (0.3-4.3) | 0.89 | 1.1 (0.3-4.0) | 0.89 | | | 1.1 (0.2-5.9) | | | 0.93 | | |  | |
| Receiving adjuvant chemotherapy (n=251) | 1.5 (0.5-5.1) | 0.49 | 1.0 (0.3-3.3) | 0.97 | | | 1.8 (0.4-7.9) | | | 0.42 | | |  | |
| Without receiving adjuvant chemotherapy (n=163) | 2.3 (0.7-7.4) | 0.15 | 1.7 (0.6-5.2) | 0.32 | | | 4.5 (1.3-15.6) | | | 0.02 | | |  | |
| **CYP3A5 rs776746 ( T/T vs. T/C+C/C)** | | |  | |  | | |  | | |  | | | |
| Total  (N=414) | 0.7 (0.2-1.9) | 0.44 | 1.2 (0.6-2.4) | 0.69 | | | 1.0 (0.3-2.7) | | | 0.92 | | |  | |
| Premenopausal  (n=251) | 0.7 (0.2-2.4) | 0.59 | 1.4 (0.6-3.2) | 0.37 | | | 1.2 (0.3-4.0) | | | 0.80 | | |  | |
| Postmenopausal  (n=163) | 0.4 (0.1-5.8) | 0.78 | 0.5 (0.1-3.9) | 0.52 | | | 0.8 (0.1-6.5) | | | 0.83 | | |  | |
| Receiving adjuvant chemotherapy (n=251) | 0.3 (0.04-2.1) | 0.22 | 0.8 (0.2-2.6) | 0.73 | | | 0.5 (0.1-3.8) | | | 0.50 | | |  | |
| Without receiving adjuvant chemotherapy (n=163) | 1.2 (0.4-4.3) | 0.75 | 1.5 (0.5-3.9) | 0.45 | | | 1.3 (0.4-4.7) | | | 0.70 | | |  | |
| **CYP2C19 rs4244285 (G/G+G/A vs. A/A)** | | |  |  | | |  | | |  | | |  | |
| Total  (N=414) | 1.6 (0.7-4.1) | 0.29 | 1.7 (0.8-4.0) | 0.19 | | | 6.4 (0.9-46.3) | | | 0.07 | | |  | |
| Premenopausal  (n=251) | 1.7 (0.5-5.7) | 0.36 | 1.8 (0.6-4.9) | 0.29 | | | 3.8 (0.5-28.1) | | | 0.20 | | |  | |
| Postmenopausal  (n=163) | 1.4 (0.3-6.3) | 0.68 | 1.7 (0.4-7.7) | 0.46 | | | - | | | - | | |  | |
| Receiving adjuvant chemotherapy (n=251) | 4.8 (0.6-35.6) | 0.13 | 3.0 (0.7-12.5) | 0.14 | | | - | | | - | | |  | |
| Without receiving adjuvant chemotherapy (n=163) | 0.9 (0.3-2.8) | 0.87 | 1.2 (0.4-3.7) | 0.72 | | | 2.7 (0.3-21.3) | | | 0.35 | | |  | |
| **CYP2C19 rs4986893 (G/G vs. G/A+A/A)** | | |  |  | | |  | | |  | | |  | |
| Total  (N=414) | 1.5 (0.7-3.5) | 0.34 | 1.4 (0.7-2.9) | 0.38 | | | 1.2 (0.5-3.1) | | | 0.67 | | |  | |
| Premenopausal  (n=251) | 1.4 (0.5-3.8) | 0.57 | 1.2(0.5-2.7) | 0.74 | | | 1.1 (0.3-3.6) | | | 0.92 | | |  | |
| Postmenopausal  (n=163) | 1.5 (0.3-6.5) | 0.62 | 1.9 (0.5-8.4) | 0.37 | | | 1.1 (0.3-5.0) | | | 0.89 | | |  | |
| Receiving adjuvant chemotherapy (n=251) | 0.9 (0.3-2.3) | 0.82 | 0.7 (0.3-1.5) | 0.35 | | | 0.5 (0.2-1.5) | | | 0.22 | | |  | |
| Without receiving adjuvant chemotherapy (n=163) | 4.1 (0.6-30.4) | 0.17 | 6.3 (0.9-46.2) | 0.07 | | | - | | |  | | |  | |
| **UGT1A1 rs4148323 (A/A+A/G vs. G/G)** | | |  |  | | |  | | |  | | |  | |
| Total  (N=414) | 1.5 (0.9-2.4) | 0.10 | 1.4 (0.9-2.1) | 0.15 | | | 1.2 (0.7-2.2) | | | 0.48 | | |  | |
| Premenopausal  (n=251) | 1.6 (0.9-2.9) | 0.13 | 1.5(0.9-2.5) | 0.13 | | | 1.0 (0.5-2.2) | | | 0.99 | | |  | |
| Postmenopausal  (n=163) | 1.1 (0.4-2.6) | 0.88 | 0.9 (0.4-2.2) | 0.85 | | | 1.4 (0.5-3.5) | | | 0.51 | | |  | |
| Receiving adjuvant chemotherapy (n=251) | 1.1 (0.6-2.1) | 0.73 | 1.1 (0.6-2.1) | 0.66 | | | 0.9 (0.4-1.9) | | | 0.72 | | |  | |
| Without receiving adjuvant chemotherapy (n=163) | 1.9 (0.9-4.2) | 0.10 | 1.5 (0.8-3.0) | 0.21 | | | 1.7 (0.7-4.2) | | | 0.22 | | |  | |
| **ABCB1_Gln rs1128503 (G/G+G/A vs. A/A)** | | |  |  | | |  | | |  | | |  | |
| Total  (N=414) | 1.7 (0.9-3.4) | 0.11 | 1.3 (0.7-2.5) | 0.42 | | | 1.6 (0.7-3.8) | | | 0.25 | | |  | |
| Premenopausal  (n=251) | 2.4 (1.1-5.3) | 0.04 | 1.7 (0.8-3.7) | 0.15 | | | 1.9 (0.6-5.9) | | | 0.24 | | |  | |
| Postmenopausal  (n=163) | 0.8 (0.2-3.5) | 0.73 | 0.6 (0.1-2.8) | 0.55 | | | 1.4 (0.3-6.9) | | | 0.71 | | |  | |
| Receiving adjuvant chemotherapy (n=251) | 1.3 (0.5-3.2) | 0.56 | 1.0 (0.4-2.4) | 0.95 | | | 1.1 (0.4-3.6) | | | 0.84 | | |  | |
| Without receiving adjuvant chemotherapy (n=163) | 3.0 (1.0-8.8) | 0.04 | 2.4 (0.9-6.4) | 0.08 | | | 2.9 (0.7-11.1) | | | 0.13 | | |  | |
| **ABCB1 rs2032582 (C/C vs. C/T+T/T)** | | |  |  | | |  | | |  | | |  | |
| Total  (N=414) | 1.3 (0.7-2.3) | 0.42 | 1.3 (0.8-2.2) | 0.31 | | | 1.9 (0.9-4.1) | | | 0.11 | | |  | |
| Premenopausal  (n=251) | 1.7 (0.8-3.6) | 0.19 | 1.8(0.9-3.6) | 0.08 | | | 3.8 (1.1-12.6) | | | 0.03 | | |  | |
| Postmenopausal  (n=163) | 0.9 (0.3-2.4) | 0.78 | 0.6 (0.2-1.5) | 0.30 | | | 0.7 (0.2-2.3) | | | 0.57 | | |  | |
| Receiving adjuvant chemotherapy (n=251) | 1.4 (0.6-3.0) | 0.41 | 1.4 (0.7-2.9) | 0.31 | | | 2.0 (0.7-5.4) | | | 0.17 | | |  | |
| Without receiving adjuvant chemotherapy (n=163) | 1.1 (0.4-2.9) | 0.87 | 1.3 (0.6-3.1) | 0.52 | | | 1.5 (0.4-5.4) | | | 0.57 | | |  | |
| **ABCB1 rs1045642 (G/G+G/A vs.A/A)** | | |  |  | | |  | | |  | | |  | |
| Total  (N=414) | 1.0 (0.5-2.0) | 0.89 | 1.0 (0.6-1.9) | 0.88 | | | 1.7 (0.7-4.3) | | | 0.26 | | |  | |
| Premenopausal  (n=251) | 1.2(0.5-2.9) | 0.66 | 1.3 (0.6-2.9) | 0.50 | | | 2.9 (0.7-12.2) | | | 0.15 | | |  | |
| Postmenopausal  (n=163) | 0.9 (0.3-2.6) | 0.90 | 0.8 (0.3-2.0) | 0.42 | | | 1.0 (0.3-3.6) | | | 0.96 | | |  | |
| Receiving adjuvant chemotherapy (n=251) | 0.9 (0.4-2.0) | 0.85 | 0.9 (0.4-2.0) | 0.90 | | | 2.4 (0.6-10.1) | | | 0.24 | | |  | |
| Without receiving adjuvant chemotherapy (n=163) | 1.6 (0.5-5.4) | 0.44 | 1.4 (0.5-4.1) | 0.50 | | | 1.2 (0.3-4.1) | | | 0.80 | | |  | |
| **ALDH3A1 rs2231142 (G/G vs. G/T+T/T)** | | |  |  | | |  | | |  | | |  | |
| Total  (N=414) | 0.7 (0.4-1.1) | 0.12 | 0.7 (0.4-1.0) | 0.05 | | | 0.7 (0.4-1.2) | | | 0.18 | | |  | |
| Premenopausal  (n=251) | 0.6 (0.3-1.1) | 0.11 | 0.5(0.3-0.9) | 0.03 | | | 0.5 (0.3-1.1) | | | 0.08 | | |  | |
| Postmenopausal  (n=163) | 0.7 (0.3-1.7) | 0.46 | 0.8 (0.4-1.8) | 0.67 | | | 0.8 (0.3-2.1) | | | 0.64 | | |  | |
| Receiving adjuvant chemotherapy (n=251) | 0.7 (0.4-1.4) | 0.33 | 0.6 (0.4-1.1) | 0.10 | | | 0.6 (0.3-1.4) | | | 0.26 | | |  | |
| Without receiving adjuvant chemotherapy (n=163) | 0.6 (0.2-1.3) | 0.17 | 0.7 (0.3-1.3) | 0.23 | | | 0.8 (0.3-2.0) | | | 0.64 | | |  | |
| **ALDH3A1 rs2228100 (G/G+G/C vs. C/C)** | | |  | |  | | |  | | |  | | |  |
| Total  (N=414) | 1.3 (0.7-2.5) | 0.35 | 1.3 (0.7-2.2) | 0.38 | | | 1.1 (0.6-2.4) | | | 0.71 | | |  | |
| Premenopausal  (n=251) | 1.3 (0.6-2.7) | 0.54 | 1.3 (0.7-2.5) | 0.45 | | | 0.9 (0.4-2.2) | | | 0.84 | | |  | |
| Postmenopausal  (n=163) | 1.2 (0.4-3.7) | 0.72 | 1.0 (0.4-2.8) | 0.93 | | | 1.8 (0.4-8.4) | | | 0.44 | | |  | |
| Receiving adjuvant chemotherapy (n=251) | 1.4 (0.6-3.3) | 0.42 | 1.0 (0.5-2.1) | 0.89 | | | 1.1 (0.4-3.0) | | | 0.82 | | |  | |
| Without receiving adjuvant chemotherapy (n=163) | 1.4 (0.5-3.6) | 0.50 | 1.9 (0.8-4.7) | 0.16 | | | 1.3 (0.4-3.9) | | | 0.70 | | |  | |
| **CYP2C9 rs1057910 (C/A vs. A/A)** | |  |  |  | | |  | | |  | | |  | |
| Total  (N=414) | 0.7 (0.2-2.1) | 0.47 | 1.0 (0.4-2.3) | 0.98 | | | 0.6 (0.1-2.3) | | | 0.42 | | |  | |
| Premenopausal  (n=251) | 0.4 (0.05-2.7) | 0.33 | 0.8 (0.2-2.5) | 0.67 | | | -- | | | - | | |  | |
| Postmenopausal  (n=163) | 1.0 (0.2-4.8) | 0.99 | 1.3 (0.4-4.8) | 0.67 | | | 1.3 (0.2-6.6) | | | 0.78 | | |  | |
| Receiving adjuvant chemotherapy (n=251) | - |  | 0.5 (0.1-3.3) | 0.44 | | | - | | | - | | |  | |
| Without receiving adjuvant chemotherapy (n=163) | 1.1 (0.3-3.8) | 0.85 | 1.4 (0.5-3.7) | 0.50 | | | 0.8 (0.2-3.7) | | | 0.78 | | |  | |
| **CYP2B6 rs4802101 (T/T vs. C/C+C/T)** | | |  | |  | | |  | | |  | | |  |
| Total  (N=414) | 2.0 (1.1-3.8) | 0.03 | 0.7 (0.4-1.3) | 0.25 | | | 1.5 (0.7-3.1) | | | 0.33 | | |  | |
| Premenopausal  (n=251) | 3.6 (1.6-7.9) | 0.002 | 0.4 (0.2-0.9) | 0.02 | | | 2.2 (0.8-6.0) | | | 0.12 | | |  | |
| Postmenopausal  (n=163) | 0.8 (0.3-2.3) | 0.64 | 1.8 (0.6-5.3) | 0.31 | | | 0.8 (0.2-2.6) | | | 0.70 | | |  | |
| Receiving adjuvant chemotherapy (n=251) | 1.9 (0.8-4.7) | 0.17 | 0.6 (0.3-1.4) | 0.24 | | | 1.7 (0.6-4.5) | | | 0.31 | | |  | |
| Without receiving adjuvant chemotherapy (n=163) | 2.1 (0.8-5.1) | 0.12 | 0.6 (0.3-1.4) | 0.24 | | | 1.1 (0.4-3.5) | | | 0.84 | | |  | |
| **CYP2B6 rs3211371 (T/C vs. C/C)** | | |  | | |  | | |  | | |  | | |
| Total  (N=414) | 19.9 (2.1-190.4) | 0.009 | 113.8 (9.9-1311.0) | 0.0001 | | | 70.2 (5.8-850.8) | | | 0.0008 | | |  | |
| Premenopausal  (n=251) | 20.1(1.7-235.8) | 0.02 | 142.4(9.6-2119.1) | 0.0003 | | | 85.0(4.6-1580.4) | | | 0.003 | | |  | |
| Postmenopausal  (n=163) | - | - | -  - |  | | | -  - | | |  | | |  | |
| Receiving adjuvant chemotherapy (n=251) | - |  | - | - | | | - | | |  | | |  | |
| Without receiving adjuvant chemotherapy (n=163) | 23.7 (1.7-322.3) | 0.02 | 143.8 (6.6-3121.4) | 0.002 | | | 381.4(11.1-13125.9) | | | 0.001 | | |  | |
| **FGFR2 rs2981582 (A/A+A/G vs. G/G)** | | |  |  | | |  | | |  | | |  | |
| Total  (N=414) | 1.6 (0.9-2.6) | 0.07 | 1.6 (1.0-2.4) | 0.05 | | | 1.9 (1.0-3.4) | | | 0.05 | | |  | |
| Premenopausal  (n=251) | 1.5(0.8-2.9) | 0.18 | 1.5 (0.9-2.6) | 0.11 | | | 2.1 (0.9-4.7) | | | 0.08 | | |  | |
| Postmenopausal  (n=163) | 1.5 (0.7-3.6) | 0.32 | 1.5 (0.7-3.3) | 0.28 | | | 1.5 (0.6-3.8) | | | 0.42 | | |  | |
| Receiving adjuvant chemotherapy (n=251) | 1.3 (0.7-2.4) | 0.42 | 1.2 (0.7-2.1) | 0.59 | | | 1.6 (0.7-3.4) | | | 0.27 | | |  | |
| Without receiving adjuvant chemotherapy (n=163) | 2.0 (0.8-4.7) | 0.12 | 2.3 (1.1-5.0) | 0.03 | | | 2.9 (1.0-8.7) | | | 0.05 | | |  | |
| **TNRC9 rs3803662 (A/A+A/G vs. G/G)** | | |  |  | | |  | | |  | | |  | |
| Total  (N=414) | 1.0 (0.5-2.0) | 0.96 | 1.3 (0.7-2.7) | 0.44 | | | 1.2 (0.5-2.8) | | | 0.74 | | |  | |
| Premenopausal  (n=251) | 0.7(0.3-1.6) | 0.41 | 1.0 (0.5-2.2) | 0.98 | | | 0.8 (0.3-2.1) | | | 0.61 | | |  | |
| Postmenopausal  (n=163) | 4.4(0.6-34.1) | 0.15 | 4.5 (0.6-33.5) | 0.15 | | | 4.2 (0.5-33.5) | | | 0.17 | | |  | |
| Receiving adjuvant chemotherapy (n=251) | 0.7 (0.3-1.6) | 0.41 | 1.0 (0.4-2.3) | 0.91 | | | 0.7 (0.2-2.3) | | | 0.61 | | |  | |
| Without receiving adjuvant chemotherapy (n=163) | 1.6 (0.5-5.3) | 0.46 | 2.2 (0.7-7.1) | 0.21 | | | 2.1 (0.5-9.5) | | | 0.32 | | |  | |
| **MAP3K1 rs889312 (C/C vs. C/A+A/A)** | | |  |  | | |  | | |  | | |  | |
| Total  (N=414) | 1.6 (1.0-2.6) | 0.04 | 1.7 (1.1-2.6) | 0.01 | | | 1.7 (0.9-2.9) | | | 0.08 | | |  | |
| Premenopausal  (n=251) | 1.9 (1.1-3.4) | 0.04 | 1.7 (1.0-2.9) | 0.04 | | | 2.2 (1.0-4.5) | | | 0.04 | | |  | |
| Postmenopausal  (n=163) | 0.8 (0.4-2.0) | 0.70 | 1.3 (0.6-2.7) | 0.53 | | | 0.8 (0.3-2.1) | | | 0.66 | | |  | |
| Receiving adjuvant chemotherapy (n=251) | 1.6 (0.9-2.8) | 0.14 | 1.8 (1.0-3.1) | 0.04 | | | 1.5 (0.7-3.2) | | | 0.26 | | |  | |
| Without receiving adjuvant chemotherapy (n=163) | 1.7 (0.8-3.8) | 0.17 | 1.7 (0.9-3.4) | 0.13 | | | 1.9 (0.8-4.7) | | | 0.16 | | |  | |
| **HCN1 rs981782 (A/A+A/C vs. C/C)** | | |  |  | | |  | | |  | | |  | |
| Total  (N=414) | 2.4 (0.7-7.7) | 0.15 | 1.3 (0.6-3.0) | 0.55 | | | 1.6 (0.5-5.3) | | | 0.43 | | |  | |
| Premenopausal  (n=251) | 4.4 (0.6-32.2) | 0.15 | 1.6 (0.5-5.3) | 0.43 | | | 2.6(0.3-19.3) | | | 0.36 | | |  | |
| Postmenopausal  (n=163) | 1.4 (0.3-6.3) | 0.65 | 1.0 (0.3-3.5) | 0.97 | | | 1.2 (0.3-5.4) | | | 0.82 | | |  | |
| Receiving adjuvant chemotherapy (n=251) | 5.2 (0.7-38.0) | 0.11 | 1.3 (0.4-3.6) | 0.66 | | | 3.4 (0.5-25.4) | | | 0.23 | | |  | |
| Without receiving adjuvant chemotherapy (n=163) | 0.9 (0.2-3.8) | 0.86 | 1.4 (0.3-6.0) | 0.66 | | | 0.7 (0.2-3.1) | | | 0.64 | | |  | |
| **5p12_2 rs10941679 (G/G vs. G/A+A/A)** | | |  |  | | |  | | |  | | |  | |
| Total  (N=414) | 0.9 (0.5-1.4) | 0.55 | 0.8 (0.5-1.3) | 0.34 | | | 0.9 (0.5-1.7) | | | 0.72 | | |  | |
| Premenopausal  (n=251) | 0.8 (0.4-1.6) | 0.62 | 0.7 (0.4-1.3) | 0.32 | | | 1.2(0.5-2.6) | | | 0.66 | | |  | |
| Postmenopausal  (n=163) | 0.6 (0.3-1.6) | 0.34 | 0.8 (0.4-1.7) | 0.48 | | | 0.4 (0.1-1.3) | | | 0.13 | | |  | |
| Receiving adjuvant chemotherapy (n=251) | 0.8 (0.4-1.6) | 0.58 | 0.8 (0.5-1.5) | 0.52 | | | 0.8 (0.3-1.7) | | | 0.52 | | |  | |
| Without receiving adjuvant chemotherapy (n=163) | 0.9 (0.3-2.2) | 0.75 | 0.7 (0.3-1.6) | 0.41 | | | 1.3 (0.5-3.4) | | | 0.66 | | |  | |
| **5p12_2 rs4415084 (T/T vs. T/C+C/C)** | | |  |  | | |  | | |  | | |  | |
| Total  (N=414) | 0.9 (0.6-1.5) | 0.71 | 0.9 (0.6-1.3) | 0.50 | | | 0.9 (0.5-1.6) | | | 0.78 | | |  | |
| Premenopausal  (n=251) | 0.9 (0.5-1.7) | 0.86 | 0.9 (0.5-1.5) | 0.57 | | | 1.1 (0.5-2.4) | | | 0.72 | | |  | |
| Postmenopausal  (n=163) | 0.7 (0.3-1.5) | 0.35 | 0.7 (0.3-1.6) | 0.44 | | | 0.6 (0.2-1.5) | | | 0.24 | | |  | |
| Receiving adjuvant chemotherapy (n=251) | 1.0 (0.5-1.8) | 0.89 | 1.0 (0.6-1.7) | 0.89 | | | 0.8 (0.4-1.7) | | | 0.58 | | |  | |
| Without receiving adjuvant chemotherapy (n=163) | 0.8 (0.3-1.9) | 0.62 | 0.7 (0.3-1.4) | 0.30 | | | 1.3 (0.5-3.3) | | | 0.64 | | |  | |
| **CYP2D6*10 vs. wt/wt (IM vs. normal)** | | |  |  | | |  | | |  | | |  | |
| Total  (N=414) | 1.1 (0.7-1.8) | 0.62 | 1.1 (0.8-1.7) | 0.51 | | | 1.5 (0.9-2.6) | | | 0.16 | | |  | |
| Premenopausal  (n=251) | 0.9 (0.5-1.6) | 0.66 | 0.8 (0.4-1.4) | 0.43 | | | 1.1(0.5-2.2) | | | 0.86 | | |  | |
| Postmenopausal  (n=163) | 2.4 (1.0-5.7) | 0.05 | 2.4 (1.0-5.7) | 0.05 | | | 2.8 (1.0-7.7) | | | 0.05 | | |  | |
| Receiving adjuvant chemotherapy (n=251) | 1.2 (0.6-2.2) | 0.59 | 1.2 (0.6-2.2) | 0.59 | | | 1.6 (0.7-3.3) | | | 0.24 | | |  | |
| Without receiving adjuvant chemotherapy (n=163) | 1.4 (0.6-3.0) | 0.40 | 1.4 (0.6-3.0) | 0.40 | | | 1.7 (0.7-4.0) | | | 0.24 | | |  | |
